# Functional dependence following intensive care unit-treated sepsis: three-year follow-up results from the prospective Mid-German Sepsis Cohort (MSC)

**DOI:** 10.1016/j.lanepe.2024.101066

**Published:** 2024-09-12

**Authors:** Carolin Fleischmann-Struzek, Sebastian Born, Miriam Kesselmeier, E. Wesley Ely, Kristin Töpfer, Heike Romeike, Michael Bauer, Sven Bercker, Ulf Bodechtel, Sandra Fiedler, Heinrich V. Groesdonk, Sirak Petros, Stefanie Platzer, Hendrik Rüddel, Torsten Schreiber, Konrad Reinhart, André Scherag

**Affiliations:** a Institute of Infectious Diseases and Infection Control, Jena University Hospital/Friedrich-Schiller-University Jena, Jena, Germany; b Integrated Research and Treatment Center, Centre for Sepsis Control and Care (CSCC), Jena University Hospital/Friedrich-Schiller-University Jena, Jena, Germany; c Institute of Medical Statistics, Computer and Data Sciences (IMSID), Jena University Hospital/Friedrich-Schiller-University Jena, Jena, Germany; d Veteran's Affairs Tennessee Valley Geriatric Research, Education and Clinical Center (GRECC), Nashville, TN, USA; e Critical Illness, Brain Dysfunction, Survivorship (CIBS) Center, Vanderbilt University Medical Center, Nashville, TN, USA; f Sepsis Foundation, Berlin, Germany; g Department of Anesthesiology and Intensive Care Medicine, Jena University Hospital/Friedrich-Schiller-University Jena, Jena, Germany; h Department of Anesthesiology and Intensive Care Medicine, University Hospital of Leipzig, Leipzig, Germany; i Department of Interdisciplinary Intensive Care Medicine and Rehabilitation, Klinik Bavaria Kreischa, Kreischa, Germany; j Center for Clinical Studies (ZKS Jena), Jena University Hospital/Friedrich-Schiller-University Jena, Jena, Germany; k Department of Interdisciplinary Intensive Care Medicine and Intermediate Care, Helios Clinic Erfurt, Health and Medical University Erfurt, Erfurt, Germany; l Medical ICU, University Hospital Leipzig, Leipzig, Germany; m Central Hospital Bad Berka GmbH, Bad Berka, Germany; n Department of Anesthesiology and Operative Intensive Care Medicine (CCM, CVK), Charité – Universitätsmedizin Berlin, Berlin Institute of Health, Berlin, Germany

**Keywords:** Sepsis, Post-Sepsis-Syndrome, Long-term outcome, Sequelae, Functional outcome

## Abstract

**Background:**

Surviving sepsis can lead to chronic physical, psychological and cognitive impairments, which affect millions of patients worldwide, including survivors after COVID-19 viral sepsis. We aimed to characterize the magnitude and trajectory of functional dependence and new impairments post-sepsis.

**Methods:**

We conducted a prospective cohort study including sepsis survivors who had been discharged from five German intensive care units (ICUs), until 36 months post-discharge. Primary outcome was functional dependence, defined as ≥1 impaired activity of daily living (ADL; 10-item ADL score <100), self-reported nursing care dependence or nursing care level. Secondary outcome was post-sepsis morbidity in the physical, psychological or cognitive domain. We used a multistate, competing risk model to address competing events in the course of dependence, and conducted multiple linear regression analyses to identify predictors associated with the ADL score.

**Findings:**

Of 3210 sepsis patients screened, 1968 survived the ICU treatment (61.3%). A total of 753 were included in the follow-up assessments of the Mid-German Sepsis cohort. Patients had a median age of 65 (Q1–Q3 56–74) years, 64.8% (488/753) were male and 76.1% (573/753) had a septic shock. Considering competing risk modelling, the probability of still being functional dependent was about 25%, while about 30% regained functional independence and 45% died within the three years post-sepsis. Patients reported a high burden of new and often overlapping impairments until three years post-sepsis. In the subgroup of three-year survivors (n = 330), new physical impairments affected 91.2% (n = 301) while new cognitive and psychological impairments were reported by 57.9% (n = 191) and 40.9% (n = 135), respectively. Patients with pre-existing functional limitations and higher age were at risk for low ADL scores three years after sepsis.

**Interpretation:**

Sepsis survivorship was associated with a broad range of new impairments and led to functional dependence in around one quarter of patients. Targeted measures are needed to mitigate the burden of this Post-Sepsis-Syndrome and increase the proportion of patients that achieve functional improvements.

**Funding:**

This work was supported by the Integrated Research and Treatment Center, 10.13039/501100013457Center for Sepsis Control and Care (CSCC) at the 10.13039/501100007653Jena University Hospital funded by the German Ministry of Education and Research and by the Rudolf Presl GmbH & Co, Kreischa, Germany.

**Research in context**

**Evidence before this study**

Sepsis survivorship is associated with a broad spectrum of long-term impairments and a decline in functional capacity. However, prospective studies on the occurrence of post-sepsis morbidity in the context of long-term survivorship considering all three domains (cognitive, psychological, and physical) of post-sepsis morbidity are scarce. We searched PubMED for peer reviewed papers published from inception to 15/08/2024 using the terms (sepsis OR septic∗) AND cohort AND (long-term OR post-sepsis) AND survivor∗ AND (function OR ADL OR “activities of daily living”). Previous cohorts defined the trajectories of general ICU survivorship, patient subgroups (e.g. older survivors, patients with malignancies) or sepsis subforms (e.g. abdominal sepsis), but only a few large-scale patient cohort sets exist that investigate long-term functional outcomes explicitly for broad sepsis survivor populations beyond the first year after sepsis. This hampers our understanding on which patients return to their baseline functional states while others remain impaired, and poses barriers on the development and implementation of effective treatment strategies, particularly for patients at risk for persistent poor long-term outcomes.

**Added value of this study**

We assessed the probability of functional dependence, as well as cognitive, psychological and physical post-sepsis impairments by self-report and standardized instruments in a prospective, multicenter cohort study of sepsis survivors. Follow-ups were conducted with low rates of drop-out among the 753 survivors. By considering the competing risk in outcomes in a multistate model with three states (functional dependence, functional independence, all-cause death), we have provided more profound estimates of the trajectories of sepsis survivors than have been available previously. Furthermore, we examined predictors for impaired functional capacity which may help to provide specific screening and aftercare for the most vulnerable groups.

**Implications of all the available evidence**

We found a considerable burden of functional dependency and post-sepsis morbidity in our survivor cohort. Psychological, cognitive, or physical impairments affected nearly all survivors under observation and often overlapped. However, we were also able to show that once functional independence was acquired, patients were likely to remain in this state. This highlights the opportunities of interdisciplinary rehabilitation and aftercare, particularly in elderly patients or patients with pre-existing functional impairments, which we found to be at highest risk for functional dependence three years after the acute disease.

## Introduction

Sepsis is a common and life-threatening disease that is estimated to affect nearly 50 million patients worldwide every year.^1^ Roughly, two out of three patients survive the acute illness under best available intensive care unit (ICU) treatment.^2^ Their recovery is often prolonged, as many survivors experience new or worsened cognitive, psychological and physical impairments. These impairments are collectively referred to as Post-Sepsis-Syndrome^3^ or Post-Intensive-Care-Syndrome (PICS) in patients with ICU treatment,^4^ with sepsis being the primary driver of PICS-related disability following critical illness.^5^ Impairments impede patients’ return to independent living for months or even years.^6^, ^7^, ^8^ They are not restricted to older survivors, but also affect previously unimpaired and younger patients,^9^ similar to what was learned by Pandharipande et al. about 20 and 30 year olds developing rapidly acquired dementia following critical illness.^7^ Data from two large clinical trials suggest that one third of previously functionally independent sepsis survivors had not returned to independent living six months later.^10^ According to a US study, elderly survivors developed on average between 1.50 and 1.57 new limitations in the activities of daily living, which were still observable at least eight years post-sepsis.^11^ This post-sepsis morbidity is extremely burdensome for patients, their relatives, and the health system as a whole. In the US, annual direct costs for sepsis and subsequent post-sepsis treatment were estimated at 67 billion US dollar for only Medicare beneficiaries.^12^ The COVID-19 pandemic, with the epidemic of acute delirium that drives PICS,^13^ has dramatically amplified this burden, as infection-associated chronic conditions (IACC) after COVID-19 and Influenza greatly overlap with the Post-Sepsis-Syndrome and PICS.^14^

To date, prospective data on the occurrence of post-sepsis morbidity in the context of long-term survivorship considering all three domains (cognitive, psychological, and physical) are scarce. While we do have cohorts that help us define the trajectories of general ICU survivorship and the development of frailty and PICS,^15^^,^^16^ only a few large patient cohort sets exist that investigate long-term functional outcomes explicitly for sepsis survivors beyond the first year after survival.^17^ We therefore lack full understanding of which patients return to their baseline functional states while others remain impaired. This hampers the development and implementation of effective treatment strategies, particularly for patients at risk for persistent poor long-term outcomes.^18^^,^^19^

With the Mid-German Sepsis Cohort (MSC), we aimed to characterize the magnitude and trajectory of functional dependence and post-sepsis morbidity considering all three domains of the Post-Sepsis-Syndrome. Here we focus on three aspects during a three year follow-up after sepsis. As primary outcome, we analyze the trajectory of functional dependence by applying multi-state modelling in the presence of the competing risk of death. As secondary outcomes, we report on post-sepsis morbidity to explore potential reasons for functional dependence and test literature-derived variables to predict functional dependence three years post-sepsis.

## Methods

This prospective cohort study was pre-registered in the German Clinical Trials Registry (DRKS00010050), approved by the institutional review board/Independent Ethics Committee of the Friedrich-Schiller-University Jena (#4669-01/16) and the responsible boards of the participating centers. The reporting followed the Strengthening the Reporting of Observational Studies in Epidemiology (STROBE) Statement.

### Study design and procedures

A detailed description of study methodology has been previously published.^20^ In short, we conducted a prospective longitudinal cohort study in intensive care units (ICUs) of five study sites in Germany (university hospitals in Jena, Halle/Saale and Leipzig, tertiary care hospitals in Bad Berka and Erfurt). Patient recruitment took place between 15th of April 2016 and 30th of November 2018. In the participating ICUs, patients were screened daily for eligibility for enrolment in the MSC. Eligibility criteria included age ≥18 years, presence of sepsis or septic shock and no prior participation in the MSC. Sepsis was defined as clinically suspected or microbiologically proven infection and presence of at least one organ dysfunction due to infection.^21^ We obtained written informed consent for participation in the follow-up assessments by patients or legal representatives. Follow-up assessments were conducted three, six, 12, 24 and 36 months ± six weeks after ICU discharge by telephone or face-to-face with patients or proxies by trained investigators. The training included a structured induction and the conduct of at least 5 joint interviews with a supervisor, as well as regular peer feedback on the conduct of the interviews during the follow-up.

### Participants

**Fig. 1:**
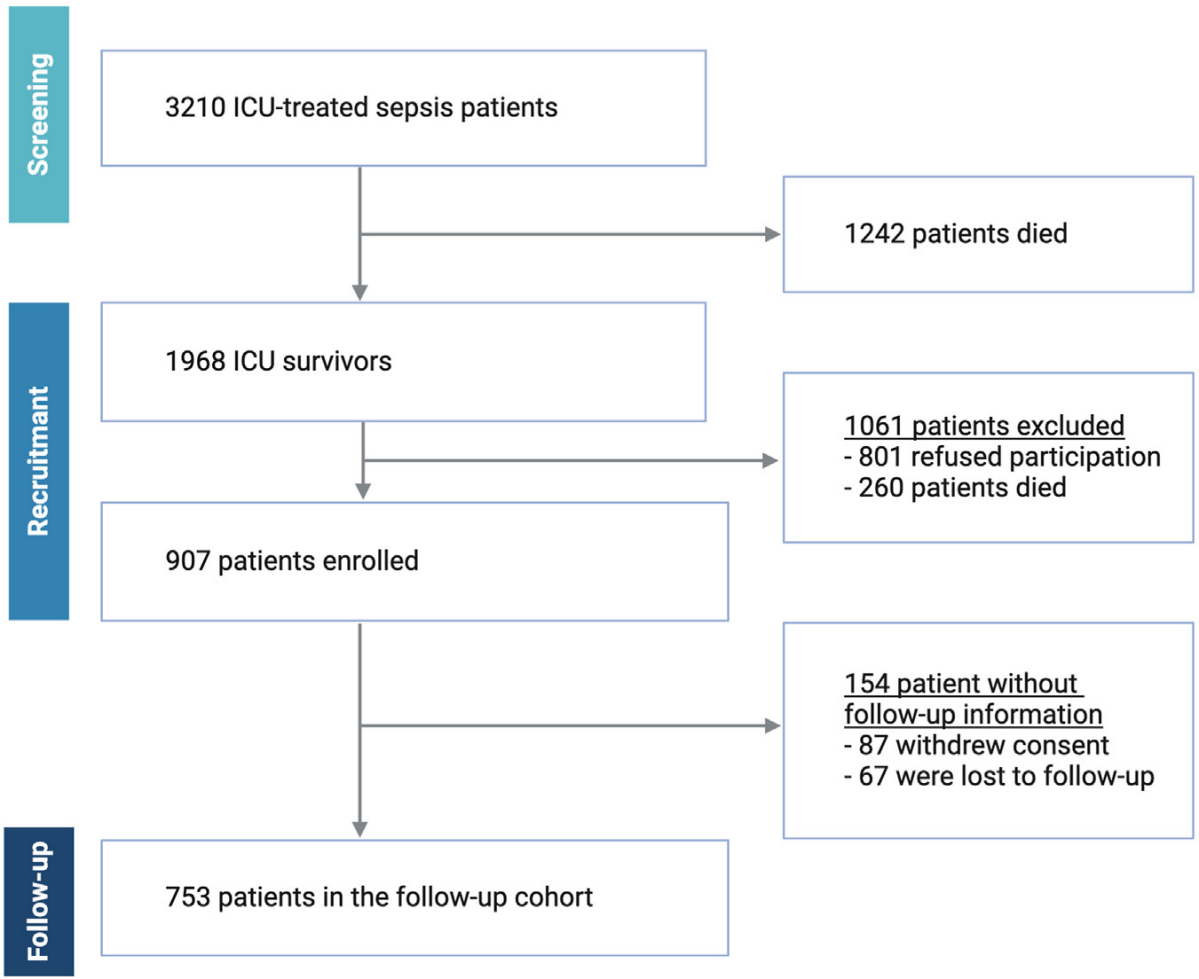
**Flow of patient inclusion**. Abbreviation: ICU, intensive care unit.

Over a 30.5-months recruitment period, we assessed 3210 ICU-treated sepsis patients in total, of whom 78.2% had septic shock. Their median maximum SOFA score was 15 (Q1, Q3 12, 18) and 80.7% received mechanical ventilation. A total of 38.7% and 47.4% of patients died until ICU and hospital discharge, respectively. Of 1968 ICU sepsis survivors, we enrolled 907 patients for follow-up in the MSC (Fig. 1). The demographic and clinical characteristics of all three patient groups (i.e. all ICU-treated sepsis patients, all ICU survivors and among them those who agreed to participate in the FU interviews) are reported in a previously published cohort profile.^22^

### Outcomes

The primary outcome was the probability of functional dependence at one- and three-years post-ICU discharge, which was defined as at least one impaired activity of daily living (ADL; 10-item ADL score <100, for information on the score, see Supplement), self-reported nursing care dependence or self-reported presence of a nursing care level (entitling for compensations for formal or informal nursing care in the German health care system). The primary outcome was assessed among patients in the follow-up cohort with at least one completed follow-up or with information on vital status after ICU discharge. As secondary outcomes, we assessed post-sepsis morbidity in the cognitive, psychological and physical domain by a list of self-reported symptoms by patients and proxies at three, six, 12, 24 and 36 months post-sepsis among patients who participated in the respective follow-up interviews. The list was developed by experts in the field of rehabilitation medicine, general medicine and intensive care of the Mid-German Sepsis Alliance based on input from sepsis survivors and their relatives. *Cognitive impairment* was defined as presence of at least one of five symptoms (impairment of attention, concentration, memory, consciousness or trouble with speech and finding words), *psychological impairment* by least one of four symptoms (feeling depressed, anxious, having nightmares, change in character), and *physical impairments* by at least one of eleven symptoms (renal impairment, respiratory dysfunction, dysphagia, muscle weakness/muscle loss, difficulties walking, paresthesia/impaired sensation, weight loss, sensory disorders, urinary incontinence, sexual disorders, pain). *Other impairments* comprised insomnia, and/or fatigue (problems to cope with everyday life). For all self-reported symptoms, we asked patients to determine the onset of symptoms as prior or post-sepsis to estimate the incidence of symptoms. Self-reported symptoms were compared with the results of validated instruments for cognitive impairment, depression, anxiety, somatization, post-traumatic stress disorder (PTSD), and fatigue (see Supplement).

### Analyses

To address competing events in the course of dependence, we used a multistate, competing risk model with three states (functional dependence, functional independence, all-cause death) to analyze the primary outcome. We assumed an underlying time-homogeneous process and assessed the 12- and 36-months transition probabilities between the three states from a state of function dependence at ICU discharge. We used three datasets for the analyses. Analysis set 1: ICU discharge until 36 months post-discharge, analysis set 2: ICU discharge until 12 months post-discharge, analysis set 3: 12 months post-discharge until 36 months post-discharge. For further definitions, see Supplement Table S1. The secondary outcome post-sepsis morbidity was analyzed descriptively and is reported for patients without missing data. Furthermore, we tested literature-derived^23^^,^^24^ predictors associated with the outcome ADL score among (a) 12-months and (b) 36-months survivors in simple and multiple linear regression analyses, and report the explained variance (R^2^) when including all predictors. The regression analyses are described in detail in Supplement. All analyses were conducted using R (version 4.0.2) with the packages msm (version 16.8),^25^ and lavaan (version 0.6–16).^26^

### Role of the funding sources

The funders of the study had no role in study design, data collection, data analysis, data interpretation, or writing of the report.

## Results

We enrolled 907 sepsis survivors for participation in the Mid-German Sepsis Cohort follow-up assessments (for the Kaplan–Meier Curve, see Supplement Figure S1). A total of 753 survivors participated in any of the follow-up assessments (590 participated in the three-months and 330 in the three-year follow-up; while 309 (34.1%) died within the three-year follow-up period). Median age at enrolment in the 753 patients was 65 (Q1–Q3 56–74) years and 64.8% of them were male (Table 1). The majority (76.1%) had a septic shock. Median maximum SOFA score during ICU treatment was 13 (Q1–Q3 10–15), 70.3% patients required mechanical ventilation, and 31.7% renal replacement therapy during their ICU treatment. Interview characteristics are provided in Supplement Table S2.

**Table 1:** Demographics and clinical features of the initial hospital stay of survivors who completed at least one follow-up assessment or for whom vital information were available in the follow-up of the Mid-German Sepsis Cohort.

Characteristic	n	Distribution
Number of patients	753	
Age, in years	753	65 (56, 74)
Male sex	753	488 (64.8%)
Comorbidities^a^	752	
Diabetes		217 (28.9%)
Chronic pulmonary disease		126 (16.8%)
Renal disease		95 (12.6%)
Congestive heart failure and myocardial infarction		161 (21.4%)
Cancer		185 (24.6%)
Dementia		15 (2.0%)
Cerebrovascular disease		59 (7.8%)
Liver disease		65 (8.6%)
HIV/AIDS		4 (0.5%)
Other		96 (12.8%)
Number of comorbidities		1 (1, 2)
Distribution		
0		175 (23.3%)
1		279 (37.1%)
2–4		278 (37.0%)
>4		20 (2.7%)
Charlson Comorbidity Index, unweighted		4 (2, 6)
Origin of infection	753	
Hospital-acquired		407 (54.1%)
Community-acquired		346 (45.9%)
Focus of infection	752	
Known		700 (93.1%)
Among them:		
Pneumonia		312 (44.6%)
Other upper or lower respiratory tract		42 (6.0%)
Intraabdominal		170 (24.3%)
Primary bacteraemia		104 (14.9%)
Urogenital		90 (12.9%)
Bones/soft tissue		55 (7.9%)
Postoperative wound infection		23 (3.3%)
Gastrointestinal		24 (3.4%)
Thoracic (empyema/mediastinitis)		27 (3.9%)
Cardiovascular		22 (3.1%)
Device-related infection		17 (2.4%)
Central nervous system		8 (1.1%)
Other		27 (3.9%)
Microbiological etiology		
Blood culture sampling	753	
Positive blood cultures		366 (48.6%)
Negative blood cultures		353 (46.9%)
No blood cultures performed		34 (4.5%)
Cultures from other sterile compartments	746	
Positive cultures		519 (69.6%)
Negative cultures		227 (30.4%)
Type of microbiologically proven infection		
Pathogens detected	745	596 (80.0%)
Among them:		
Bacterial pathogens		572 (96.0%)
Fungal pathogens		107 (18.0%)
Viral pathogens		13 (2.2%)
Presence of multi-resistant pathogens	743	122 (16.4%)
Among them:		
Gram-positive bacteria		51 (41.8%)
Gram-negative bacteria		73 (59.8%)
Unknown		5 (4.1%)
Organ dysfunctions at sepsis onset	753	
Arterial hypoxemia		533 (70.8%)
Renal dysfunction		384 (51.0%)
Metabolic acidosis		360 (47.8%)
Acute encephalopathy		156 (20.7%)
Thrombocytopenia		174 (23.1%)
Septic shock		573 (76.1%)
Among them:		
Septic shock patients with >2.0 mmol/l at sepsis onset		350 (61.1%)
Number of organ dysfunctions		3 (2, 4)
Distribution:		
0		0 (0.0%)
1		93 (12.4%)
2		207 (27.5%)
>2		453 (60.2%)
Presence of delirium during ICU stay	752	243 (32.3%)
Days with presence of delirium		4 (2, 9)
Vasopressor therapy during ICU stay	753	605 (80.3%)
Organ replacement or support therapy during ICU stay		
Mechanical ventilation	751	528 (70.3%)
ECMO or other lung replacement therapy	752	15 (2.0%)
Renal replacement therapy	747	237 (31.7%)
Other replacement therapy	751	4 (0.5%)
Maximal SOFA score during ICU stay	692	13 (10, 15)
Length of ICU stay, in days	753	10 (4, 25)
Length of hospital stay, in days	753	35 (22, 52)
Tracheostomy at hospital discharge	694	106 (15.3%)
Ventilation at hospital discharge	694	47 (6.8%)
Dialysis at hospital discharge	694	64 (9.2%)
Discharge to	690	
Home		360 (52.2%)
Rehabilitation facility		175 (25.4%)
Transfer to acute care hospital		121 (17.5%)
Nursing home		20 (2.9%)
Other		14 (2.0%)

For each characteristic, the number of patients with information on this characteristic (n) as well as the distribution within these patients (median with first and third quartile (Q1, Q3) or absolute and relative frequencies) are provided. For several characteristics, multiple answers per patient were possible. Abbreviations: DNR, do not resuscitate; ECMO, extracorporeal membrane oxygenation; ICU, intensive care unit; MSC, Mid-German Sepsis Cohort; SOFA, sequential organ failure assessment, which was assessed at least once a day during ICU stay.

a If individual items were not documented, they were considered as not existent. Patients with no documentation were excluded.

### Primary outcome: functional dependence over a period of three years after sepsis

**Fig. 2:**
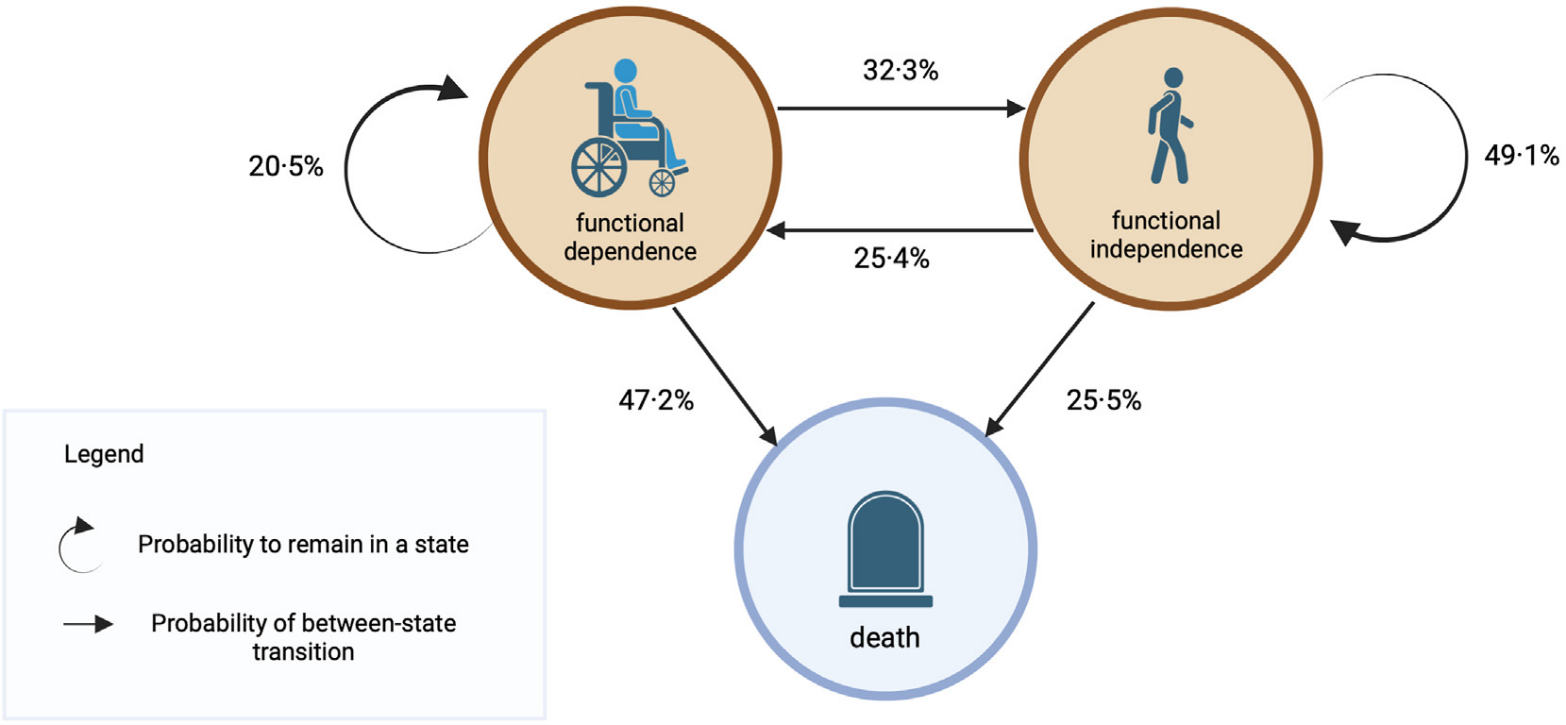
**Transition probabilities three years after discharge from the intensive care unit (ICU)**. Results from the multistate, competing risk modelling for 753 patients (analysis set 1) of the Mid-German Sepsis Cohort with available information on their state of dependence during follow-up. Individuals were considered to be in a dependent state at the time of discharge from the ICU. Percentages in the round circles indicate the probability to remain in the respective state and percentages at arrows indicate the probability for the respective transition within the three-year time frame. Figure created with BioRender.com.

Considering competing risk modelling from hospital discharge to three-year post-discharge assessment (analysis set 1, n = 753 patients), the probability was 20.5% to remain functionally dependent within the three years after surviving sepsis (Fig. 2). Within the same period, the probabilities were 32.3% to regain functional independence and 47.2% to die. Transitions between states occurred with higher probabilities within the first 12 months after sepsis (Supplement Figure S2). Patients were highly likely to remain in that state three years after ICU discharge once they were in either a state of functional dependence or independence (probability to remain independent: 71.2%, probability to remain dependent: 59.0%, analysis set 3, n = 429 patients, Supplement Figure S3). Summarized across analysis sets 1 to 3, approximately 25% of sepsis survivors remained functionally dependent, 30% returned to functional independence and 45% died within three years post-sepsis. Further details on the modelling can be found in the Supplement (Supplement Figures S4–S7).

### Secondary outcomes: post-sepsis morbidity among three-year survivors

**Fig. 3:**
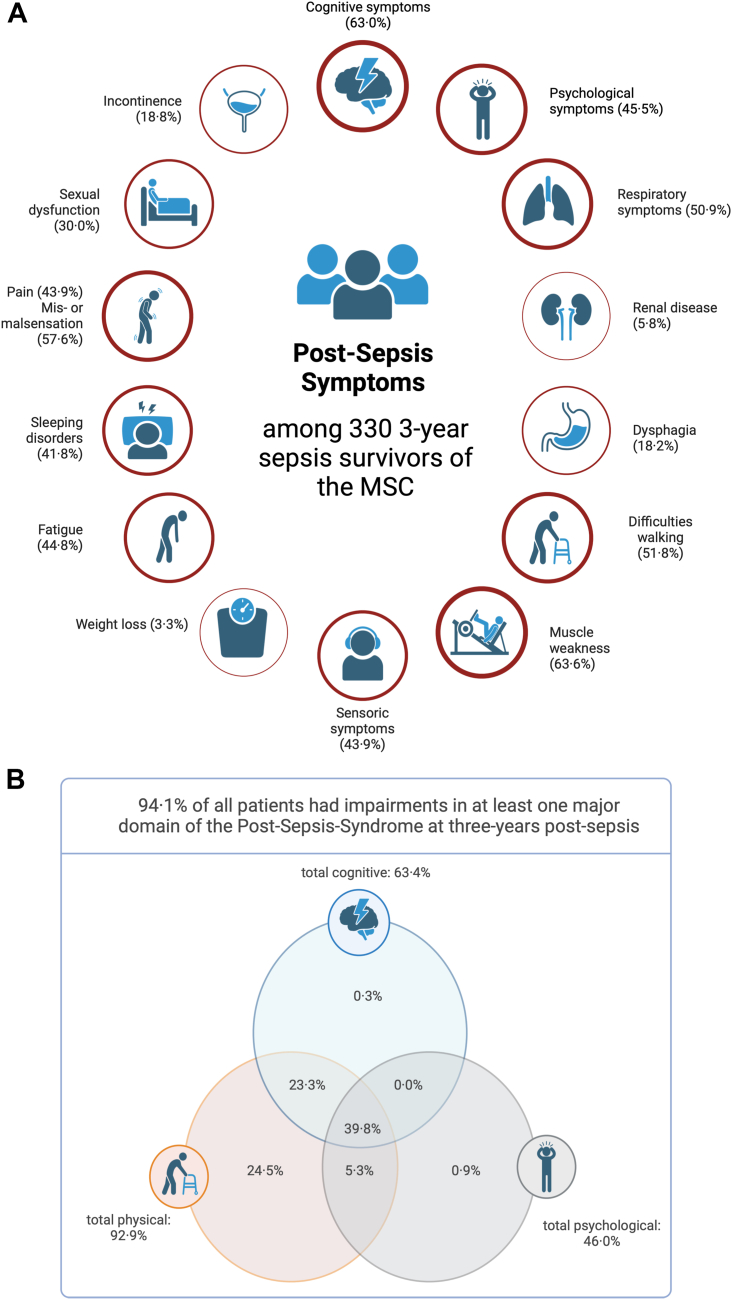
**A: Post-Sepsis symptoms as reported by the 330 three-year survivors of the Mid-German Sepsis Cohort (MSC)**. Percentages indicate the proportion of individuals affected by the self-reported symptoms. Figure created with BioRender.com. **B: Overlap in self-reported cognitive, psychological and physical impairments among 322 three-year survivors of the Mid-German Sepsis Cohort with complete information on all three domains**. Percentages indicate the proportion of individuals affected by the self-reported impairments. Figure created with BioRender.com.

A total of 92.1% of three-year survivors (304 out of 330) reported prevalent physical symptoms three years post-sepsis; and 91.2% reported the presence of physical symptoms that *newly* occurred after sepsis (Table 2). Muscle weakness/loss, mis- and malsensation and walking difficulties were the most common physical symptoms occurring in 63.6%, 57.6% and 51.8% of three-year survivors, respectively (Fig. 3A).

**Table 2:** Self-reported prevalent and new onset impairments of sepsis survivors at the one-year and three-year follow-up assessment.

Time post-sepsis	1 year (N = 459)				3 years (N = 330)			
self-reported impairment	prevalent		new onset		prevalent		new onset	
	n	% [95% CI]	n	% [95% CI]	n	% [95% CI]	n	% [95%-CI]
**I. Cognitive**	264	57.5 [53.0, 62.0]	218	47.5 [43.0, 52.1]	208	63.0 [57.7, 68.1]	191	57.9 [52.5, 63.1]
**II. Psychological**	226	49.2 [44.7, 53.8]	203	44.2 [39.7, 48.8]	150	45.5 [40.2, 50.8]	135	40.9 [35.7, 46.3]
**III. Physical**	421	91.7 [88.8, 93.9]	408	88.9 [85.7, 91.4]	304	92.1 [88.7, 94.6]	301	91.2 [87.7, 93.8]
Sensoric symptoms	172	37.5 [33.2, 42.0]	95	20.7 [17.2, 24.6]	145	43.9 [38.7, 49.3]	90	27.3 [22.7, 32.3]
Renal disease	39	8.5 [6.3, 11.4]	19	4.1 [2.7, 6.4]	19	5.8 [3.7, 8.8]	12	3.6 [2.1, 6.2]
Respiratory symptoms	207	45.1 [40.6, 49.7]	107	23.3 [19.7, 27.4]	168	50.9 [45.5, 56.3]	119	36.1 [31.1, 41.4]
Dysphagia	69	15.0 [12.1, 18.6]	58	12.6 [9.9, 16.0]	60	18.2 [14.4, 22.7]	48	14.5 [11.1, 18.8]
Muscular weakness	261	56.9 [52.3, 61.3]	230	50.1 [45.6, 54.7]	210	63.6 [58.3, 68.6]	193	58.5 [53.1, 63.7]
Walking difficulties	211	46.0 [41.5, 50.5]	164	35.7 [31.5, 40.2]	171	51.8 [46.4, 57.2]	153	46.4 [41.1, 51.8]
Mis- or malsensation	251	54.7 [50.1, 59.2]	179	39.0 [34.6, 43.5]	190	57.6 [52.2, 62.8]	146	44.2 [39.0, 49.6]
Weight loss	34	7.4 [5.3, 10.2]	32	7.0 [5.0, 9.7]	11	3.3 [1.9, 5.9]	10	3.0 [1.7, 5.5]
Incontinence	81	17.6 [14.4, 21.4]	63	13.7 [10.9, 17.2]	62	18.8 [14.9, 23.4]	56	17.0 [13.3, 21.4]
Sexual dysfunction	114	24.8 [21.1, 29.0]	69	15.0 [12.1, 18.6]	99	30.0 [25.3, 35.2]	83	25.2 [20.8, 30.1]
Pain	201	43.8 [39.3, 48.4]	113	24.6 [20.9, 28.8]	166	43.9 [38.7, 49.3]	118	35.8 [30.8, 41.1]
**IV. Other impairments**	276	60.1 [55.6, 64.5]	211	46.0 [41.5, 50.5]	203	61.5 [56.2, 66.6]	161	48.8 [43.4, 54.2]
Fatigue	174	37.9 [33.6, 42.4]	143	31.2 [27.1, 35.5]	148	44.8 [39.6, 50.2]	122	37.0 [31.9, 42.3]
Sleeping disorders	197	42.9 [38.5, 47.5]	118	25.7 [21.9, 29.9]	138	41.8 [36.6, 47.2]	85	25.8 [21.3, 30.7]

%, proportion of patients reporting the respective impairment; CI, confidence interval; N, number of patients included in the assessment; n, number of patients reporting the respective impairment.

A total of 63.0% of three-year survivors (208 out of 330) had cognitive symptoms and 45.5% were affected by psychological symptoms (Table 2). New onset cognitive and psychological symptoms were present in 57.9% and 40.9% of three-year survivors, respectively. Sleep disorders were reported by in 41.8% of three-year survivors (new onset sleep disorders: 25.8% of patients). Fatigue affected 44.8% of survivors (new onset fatigue: 37.0%). Of note, symptoms were more often self-reported by survivors than detected as clinically relevant by questionnaires (Supplement Table S3, cognitive symptoms: 63.0% by self-report vs. 21.5% by instruments T-MoCA or IQCODE, psychological symptoms: 45.5% by self-report vs. 10.2% by BSI-18 (anxiety, depression, somatization) or 10.0% by PTSS-10 (PTSD)). The only exception was fatigue. A total of 44.8% of three-year survivors reported fatigue symptoms, but 75.8% (248/327) had a Chalder Fatigue Score indicating clinically relevant fatigue symptoms (Supplement Table S3). For data of other follow-up assessments, see Supplement Tables S4 and S5.

Impairments often overlapped in more than one domain (Fig. 3B) and 39.8% of three-year survivors had impairments in all three domains. The trajectories of physical, cognitive, and psychological symptoms with and without onset after sepsis in the subgroup of three-year survivors showed differential pattern (Supplement Tables S6 and S7).

While self-reported cognitive symptoms markedly increased between three months and three years post-sepsis (+16.6 percentage points), psychological and physical symptoms remained relatively stable (+1.0 and −2.9 percentage points, respectively, Supplement Table S6). Trends for new onset impairments were similar (Supplement Table S7). On the other hand, instruments mapped a declining prevalence of cognitive and psychological impairments (−7.3 percentage points for cognitive symptoms by IQCODE or T-MoCA, −5.1 percentage points for psychological symptoms according to BSI-18 and PTSS-10, respectively, between three-months and three-year follow-up).

### Literature-derived predictors of functional dependence (ADL scores)

Results of the simple regression models are shown in Supplement Figure S8. In the multiple linear regression model including 330 three-year sepsis survivors, we found that higher pre-sepsis ADL scores and chronic cerebrovascular disease were positively associated with the three-year ADL score, while age was negatively associated with the three-year ADL score (Table 3). We observed no evidence for associations between the ADL score and sex, maximum SOFA score, ICU length of stay, number of organ replacement therapies, pre-existing renal, cardiovascular, respiratory, liver diseases, cancer, and the discharge to rehabilitation. The full model with all predictors explained 24.5% of the variance in the three-year ADL score. For the outcome one-year ADL score (Table 3), the results for age and pre-sepsis ADL score were similar, but negative associations were existent with ICU length of stay and dementia. No further evidence for associations could be observed. The explained variance in one-year ADL score was 29.4% for the model with all predictors.

**Table 3:** Predictors of activities of daily living (ADL) score at one- and three-year post-sepsis assessed in one- and three-year sepsis survivors from the multiple linear regression analysis.

Predictors [Reference category or step size]	1 year post-sepsis (n = 459)			3 years post-sepsis (n = 330)		
	β	95% CI for β	p-value	β	95% CI for β	p-value
Age	−0.152	[−0.235, −0.069]	<0.001	−0.186	[−0.285, −0.088]	<0.001
Sex [male]	−0.060	[−0.139, 0.020]	0.142	−0.022	[−0.118, 0.075]	0.656
Maximum SOFA score	−0.046	[−0.143, 0.052]	0.358	−0.039	[−0.155, 0.077]	0.508
Renal failure [absence]	−0.053	[−0.133, 0.027]	0.195	−0.015	[−0.110, 0.081]	0.760
Chronic cardiovascular disease [absence]	−0.010	[−0.090, 0.071]	0.813	−0.027	[−0.126, 0.071]	0.585
Cancer [absence]	0.030	[−0.051, 0.110]	0.470	−0.003	[−0.103, 0.097]	0.950
Chronic cerebrovascular disease [absence]	0.011	[−0.069, 0.090]	0.794	0.101	[0.005, 0.197]	0.039
Liver disease [absence]	0.066	[−0.014, 0.146]	0.104	0.009	[−0.088, 0.106]	0.854
Chronic respiratory disease [absence]	0.028	[−0.052, 0.107]	0.493	0.051	[−0.047, 0.149]	0.306
Dementia [absence]	−0.086	[−0.166, −0.007]	0.033	–	–	–
Pre-sepsis ADL score	0.454	[0.381, 0.528]	<0.001	0.457	[0.371, 0.543]	<0.001
Number of organ replacement therapies	−0.034	[−0.134, 0.067]	0.510	−0.068	[−0.191, 0.055]	0.277
Length of ICU stay	−0.153	[−0.251, −0.055]	0.002	−0.095	[−0.215, 0.025]	0.120
Discharge to rehabilitation [absence]	−0.056	[−0.143, 0.032]	0.211	−0.034	[−0.143, 0.075]	0.538

Predictors were assessed at the index ICU stay except for discharge disposition (assessed at hospital discharge) and pre-sepsis ADL score (assessed at three-months follow-up). We treated ADL score (range 0–100), age and maximum SOFA score (range 0–24), number of organ replacement therapies, length of ICU stay as metric and the remaining variables as binary. β = standardized regression coefficients, CI = confidence interval. n = number of analyzed survivors. For three-year post-sepsis, dementia was a constant because none of the included sepsis survivors suffered from dementia. Reading example: The standardized regression coefficient β for the predictor “pre-sepsis ADL score” corresponds to the change in the ADL score, controlling for all other predictors, if the pre-sepsis ADL score would change by one standard deviation.

## Discussion

Our large, prospective, cohort study incorporates three-year outcomes to meet the driving unmet need to understand three foci related to the epidemiology of the Post-Sepsis-Syndrome. First, we demonstrate that of 100 ICU sepsis survivors, approximately 25 remained functionally dependent, 30 returned to functional independence and 45 died in the three years post-sepsis. In other words, of those that survived three years after sepsis, half of them are at risk to experience prolonged functional dependence. Second, almost all three-year survivors reported new symptoms after sepsis. Physical impairments affected 90 out of 100 three-year survivors, while cognitive and psychological impairments were found in 60 and 40 of 100 survivors, respectively. Overlap of impairments was common and increased over time. Third, we showed that pre-existing functional dependence and higher age increased the risk of functional dependence three years after sepsis.

This burden of long-term mortality, morbidity and functional dependence adds to the significant hospital and ICU mortality we observed in the Mid-German Sepsis cohort (38.7% and 47.4%, respectively).^22^ This underlines the acute and persistent impact of sepsis on the functional capacity of survivors and that previously impaired patients are at particular risk for not regaining functional dependence after the acute disease. Our results are in line with previous research that found that functional limitations affected a majority of sepsis survivors,^6^^,^^9^ that impairments lasted for ≥12 months post-sepsis^11^ and that the pre-sepsis functional status is a major predictor of post-sepsis functional outcome.^27^

Our study also contributes new evidence about the spectrum of post-sepsis long-term morbidity. In the three years post-sepsis, nearly all survivors are affected by heterogeneous cognitive, physical, and psychological symptoms, with symptoms co-occurring in all three domains in about 40% of three-year survivors. This is much higher than the proportion previously estimated based on German national health insurance data, in which co-occurring *new* impairments were found in between 5.2% (0–12 months post-sepsis) and 1.5% (25–36 months post-sepsis) of sepsis hospital survivors after ICU-treatment.^9^

Of note, the proportion of patients that reported subjective symptoms with onset after sepsis even increased over time in some domains in our study, most prominently in the cognitive domain. This may also indicate age effects in the elderly population of sepsis survivors and an overlap with geriatric conditions to some degree.^28^ However, previous research found that cognitive decline and functional deterioration were significantly more common among sepsis survivors compared to non-sepsis hospital controls, who developed 0.43–0.48 new limitations post-discharge (compared to 1.50–1.57 in sepsis patients).^11^ In more severe ill populations, there was no evidence for a difference between sepsis and non-sepsis survivors in terms of functional capacity.^29^ Future research may therefore focus on the pathogenesis of impairments and how sepsis compared to other factors contributes to their development.

Interestingly, we could compare self-reported symptoms against results of objective instruments. Patients reported subjective symptoms more frequently than detectable as clinically relevant by objective instruments, and both measures showed diverging temporal trends. This may be inherent to the lower threshold of reporting single symptoms compared to the detection of clinically relevant diseases by objective instruments. This finding and interpretation fits to observations in COVID-19 survivors. Here, only a small proportion of the subjectively perceived cognitive symptoms by survivors could be objectified by neurocognitive testing according to a recent study.^30^ In functional magnetic resonance imaging scans, however, patients with subjective cognitive symptoms showed compensatory neural processes with increased usage of alternate brain regions, as well as reorganized networks, for a normal performance in working memory tasks.^30^ Although the type of pathogen may have specific impact on long-term sequelae of infections and sepsis, long-term sequelae after both sepsis and COVID-19 may have in common that in both conditions, the subjectively perceived impairments can be burdensome for patients, make it difficult or impossible to return to one's previous life^31^ and require structured follow-up and aftercare for the affected patients and their relatives.^32^ Simple tools like our list of self-reported symptoms can help to capture these subjective impairments. Such tools may also be relevant as patient-reported outcome measures to be considered in clinical trials in addition to objective measures. Furthermore, it may underline the need to intensify research to better understand these discrepancies and potentially develop new diagnostic approaches. To this end, it can be beneficial to focus on the similarities between post-COVID and post-sepsis illnesses, which overlap in many domains^14^ and are still too little known at a health policy level.

In addition, our results highlight that patients with pre-existing functional dependence as well as elderly patients are at increased risk of enduring functional dependence. In contrast, the impact of the initial severity of the disease (as mirrored by the SOFA score) may be less important for long-term functional dependence. For these risk groups, we hypothesize that targeted post-sepsis care and rehabilitation may reduce the burden of long-term sequelae, e.g. the prevention and adequate management of cerebrovascular diseases, early sepsis recognition, optimal treatment including the ABCDEF-bundles^33^^,^^34^ and recovery-oriented care practices, including follow-up scheduling, receipt of functional and mental health status evaluations, sepsis education and medication optimization.^35^ Such recovery-oriented care practices have so far been only implemented incompletely according to the results of a recent survey of 26 US veterans affairs hospitals, with only 20% of patients receiving sepsis education and 55% scheduled for follow-up within two weeks.^36^ Furthermore, patients can benefit from rehabilitation,^37^^,^^38^ early home care and outpatient follow-up,^39^ as well as treatment bundles including structured hospital to outpatient care transitions, post-discharge medication review, evaluation for new impairments or symptoms, monitoring of comorbidities, and focus on care alignment, which were shown to effectively reduce mortality and readmissions in sepsis survivors.^40^ Advanced care planning may be especially important given the high long-term mortality of patients. Patients may also benefit from specific structures for post-sepsis care, e.g. in post-ICU clinics run by intensivists, which can offer tailored multidisciplinary care for the broad spectrum of post-sepsis impairments.^41^ However, such structures may not be sufficient for the large number of survivors affected. It may be important to include other stakeholder like the patients’ health care providers like general practitioners.^32^

With its comprehensive longitudinal assessments and evaluation including competing risk modeling, the Mid-German Sepsis cohort creates novel insights on post-sepsis sequelae and may help to inform patients, relatives, health care providers and politics about the patterns of functional dependence and post-sepsis morbidity. Further strengths include the multicenter cohort design, and the comprehensive clinical characterization of cohort participants and non-participants.^22^

Our study has also important limitations to consider. First, our cohort consists of sepsis survivors from five German ICUs of mainly university or tertiary care hospitals, thus the results may not be fully transferable to other survivor cohorts, including cohorts of non-ICU treated sepsis patients. One aspect in which this becomes apparent, for example, is the high median SOFA score and high proportion of patients with septic shock compared to other sepsis cohorts.^42^^,^^43^ This must also be taken into account when comparing our results with other cohort studies. Second, even though extensive efforts were made to minimize drop-out, only about half of the three-year survivors took part in the interviews. This proportion is similar or higher than in comparable cohort studies with long-term follow-up.^44^^,^^45^ However, it may still have led to bias, although the comparison of demographic and clinical characteristics shows rather small differences between the cohort of interviewed and non-interviewed patients. Third, although we planned for face-to-face assessment, we had to conduct most interviews by telephone due to the restrictions during the COVID-19 pandemic. As a consequence, we could not run tests such as handgrip strength measurements on a larger proportion of patients. Furthermore, we included both interviews with patients and/or proxies, which is why we cannot rule out proxy response bias in our data.^46^ Fourth, we did assess a list of subjective symptoms at every follow-up assessment to determine new onset and prevalent symptoms after the acute septic insult. This list mirrors the burden of perceived impairments by survivors. The discrepancies between the results of the validated instruments and subjective symptoms can themselves be seen as findings of the study and require further clarification in follow-up studies. Fifth, due to little knowledge on the course of regaining functional independence post-sepsis, we assumed a time homogenous process and did not apply multiple imputation in case of missed follow-up interviews in the multistate modelling. Sixth, analyzing predictors for the one and three-year ADL score, we focused on discharge to rehabilitation, as we lack precise information on other therapies delivered at home or at a nursing care facility, which can nevertheless have a relevant influence on the outcome of patients.

### Conclusion

Sepsis can be considered a sentinel event in the lives of affected patients, with long-lasting new symptoms and functional decline in survivors manifested in the public health problem of PICS and IACC. The broad spectrum of sequelae and the magnitude of survivors affected even three years after the septic insult underline the substantial impact sepsis has on patient morbidity and functional dependence. For those who do regain functional independence in the course of recovery, they are likely to remain in this state. Therefore, targeted measures are needed to mitigate the disease burden and increase the proportion of patients that achieve such an improvement in their condition, including more recovery-oriented care during the acute hospitalization as well as the provision of specialized, multidisciplinary aftercare in post-ICU survivorship clinics as well as structured cognitive, physical and psychological rehabilitation programs for patients affected by IACC in general.

## Contributors

AS and KR acquired funding and designed the Mid-German Sepsis cohort. CFS, UB and EWE contributed to the design. SF and SPl managed the study and were responsible for data management. SB, SPe, HVG, TS, MBu, MBa and HR led the study centers Leipzig, Erfurt, Bad Berka, Halle and Jena, and coordinated recruitment. CFS, KT and HR performed the follow-up assessments. AS, SB and MK designed the statistical analyses. SB and MK conducted the analyses. CFS, MK, SB and AS drafted the manuscript. All authors revised the manuscript for important intellectual content.

## Data sharing statement

Data access to the final cleaned data set is provided to all project applicants (primarily within the MSC investigator group) along with written use and access rules of the CSCC of the Jena University Hospital which include a brief proposal including a sketch indicating the envisaged analysis project and an additional ethical or data protection vote depending on the type of project. To ensure confidentiality, data distributed to project applicants will be double pseudonymised and any directly identifying patient information will not be provided. Due to data economy and subsequently data protection, only the variables required for the analysis project will be provided.

## Declaration of interests

Over the past 5 years, Sven Bercker was investigator in studies sponsored by BioMeriéux, Boehringer Ingelheim, AM-Pharma B.V., Roche, Takeda, Dompé, and he is member of a DSMB for a study sponsored by hemotune AG. He has not received any personal fees for lectures or as a consultant or any reimbursement of travel expenses outside the sponsored studies. Konrad Reinhart holds shares from InflaRx NV, which is based in Jena, Germany and listed at NASDQ. This company recently received emergency use authorization by the FDA for an antibody against C5a—Gohibic (vilobelimab)—to treat critically ill COVID-19 patients, which fulfill the criteria for viral sepsis. E. Wesley Ely received NIH/VA Grant support. Over the last 36 months, the institution of Heinrich Groesdonk has received fees for his lectures, for his consultant activities, for his work in an advisory board or reimbursement for personal travel expenses by Edwards Lifescience, Amomed, and he personally holds shares from Fresenius SE. All other authors declare no conflict of interest.
