# Supplementary material for: Functional dependence following intensive care unit-treated sepsis: three-year follow-up results from the prospective Mid-German Sepsis Cohort (MSC)
Source: Lancet Reg Health Eur. 2024 Sep 12;46:101066. doi: 10.1016/j.lanepe.2024.101066 (PMC11415812; doi:10.1016/j.lanepe.2024.101066)
Supplement: Supplement [file mmc1.docx]

# Supplement

# Fleischmann-Struzek C, Born S, Kesselmeier M, et al. Functional dependence after intensive care unit-treated sepsis: results from three-year follow-up of the prospective Mid-German Sepsis Cohort (MSC)

## Content

[Supplement: Methods 3](#_Toc174567767)

[Validated questionnaires used in the Mid-German Sepsis Cohort 3](#_Toc174567768)

[Multistate, competing risk modelling 3](#_Toc174567769)

[Regression analyses 5](#_Toc174567770)

[Supplement: Figures 7](#_Toc174567771)

[Figure S1: Kaplan-Meier estimate of overall survival 7](#_Toc174567772)

[Figure S2: Transition probabilities within one year after discharge from the intensive care unit (ICU) 8](#_Toc174567773)

[Figure S3: Transition probabilities within one to three years after discharge from the intensive care unit (ICU) 9](#_Toc174567774)

[Figure S4: Observed and expected prevalence of the three states (1: independence, 2: dependence, 3: all-cause death) of the multistate, competing risk model 10](#_Toc174567775)

[Figure S5: Observed and expected prevalence of the three states (1: independence, 2: dependence, 3: all-cause death) of the multistate, competing risk model 11](#_Toc174567776)

[Figure S6: Observed and expected prevalence of the three states (1: independence, 2: dependence, 3: all-cause death) of the multistate, competing risk model 12](#_Toc174567777)

[Figure S7: Fitted survival from the two transient states (independence, dependence) of the multistate, competing risk models 13](#_Toc174567778)

[Supplement: Tables 14](#_Toc174567779)

[Table S1: Overview of included data for multistate, competing risk modelling and the related time points after intensive care unit (ICU) discharge, for which the transition probabilities are estimated 14](#_Toc174567780)

[Table S2: Interview characteristics 15](#_Toc174567781)

[Table S3: Impairments post-sepsis identified by validated questionnaires for all time points of MSC follow-up (FU) assessments (longitudinal perspective) 16](#_Toc174567782)

[Table S4: Prevalent symptoms reported by interviewees for all time points of MSC follow-up assessments – cross-sectional perspective 17](#_Toc174567783)

[Table S5: New onset symptoms reported by interviewees for all time points of MSC follow-up assessments – cross-sectional perspective 18](#_Toc174567784)

[Table S6: Prevalent symptoms reported by three-year follow-up patients (N = 330) for all time points of MSC follow-up assessments – longitudinal perspective 20](#_Toc174567785)

[Table S7: New onset symptoms reported by three-year follow-up patients (N = 330) for all time points of MSC follow-up assessments – longitudinal perspective 22](#_Toc174567786)

[Table S8: Predictors of activities of daily living (ADL) score at one- and three-year post-sepsis assessed in one- and three-year sepsis survivors from the simple linear regression analysis 24](#_Toc174567787)

# Supplement: Methods

## Validated questionnaires used in the Mid-German Sepsis Cohort

Functional dependence was assessed with the Barthel Index for Activities of Daily Living (ADL).^1^ The ADL include ten items with two to three answer options (unable, needs help, independent), which rate a person’s ability to function independently regarding feeding, bathing, grooming, dressing, patients’ bowel and bladder control, toilet use and transfers (e.g. bed to chair). A sum score is calculated, with higher scores indicating a higher degree of independence. According to Depperman et al. (2008)^2^ the functional dependency based on the ADL score can be differentiated in severe (≤ 30 points), moderate (35–80 points), mild (85-95 points) and no dependency (100 points). For the assessment of cognitive impairments, we used the T-MoCA (administrated for patients) or IQCODE (administered for proxies), for which a T-MoCA score^3^ of <18 points or an IQCODE score^4^ of <23 points was considered as clinically meaningful. For psychological impairments, PTSS-10 and BSI-18 were administered, with a PTSS-10 score of <19 points^5,6^ indicating clinically relevant symptoms of post-traumatic stress disorder (PTSD) and a BSI-18 t-score^7^ <63 points indicating depression, anxiety or somatization symptoms. For fatigue, the Chalder Fatigue Score was used, with a score <4 indicating clinically relevant fatigue symptoms.^8^ Furthermore, we assessed the health-related quality of life by EQ-5D-3L^9^ and long-term all-cause mortality. Only instruments with complete data were included in the analyses.

## Multistate, competing risk modelling

To address competing events in the course of dependency from ICU discharge to the 36-months follow-up, we used a multistate, competing risk model with three states and assumed an underlying time-homogeneous process.

**Definition of the states and allowed transitions.** We considered two transient states (functional independence, dependence) and one absorbing state (death from any reason). Functional independence of an individual was defined as full ADL score (100 points) and neither self-reported nursing care dependency nor self-reported presence of a nursing degree. Consequently, an individual was dependent if the individual did not reach a full ADL score or reported the requirement of nursing care dependency or the presence of a nursing degree. Allowed transmissions were those between transient states and from a transient into an absorbing state.

**Definition of time (points).** By design, the informative sampling times (ICU discharge, five follow-up assessments scheduled at three, six, 12, 24 and 36 months after ICU discharge) were fixed, although deviations from the schedule occurred in practice. All three states were possible outcomes of the follow-up assessments, but by design at ICU discharge only the states “functional independence” and “dependence” were possible. We consider the time at which a state was observed as days since ICU discharge. We assume that the states are unknown between observation times, i.e. the state at time of the follow-up assessment was a "snapshot" of the process. In case of death, the date of death was taken as exact transition time, if documented (otherwise, the date of the following follow-up assessment was considered as observation time). For all other completed follow-up assessments, we considered the date at which the follow-up interview was conducted as observation time. In case a follow-up interview was not completed and the interview/death date was missing, the hypothetical (i.e. scheduled) time point for the respective interview was used instead.

**Analysis sets.** In our analysis set 1, we considered follow-up interviews from ICU discharge until 36 months after ICU discharge. This analysis set comprised participants with information on two or more time points. Missed follow-up interviews were excluded from analysis of the respective participant. In case of missing state information at ICU discharge for a participant, the state was set to “dependence”. From a medical point of view, this assumption is plausible as all participants were discharged from ICU after overcoming sepsis or septic shock. We relied on two further analysis sets (analysis sets 2 and 3). Follow-up interviews within one year after ICU discharge (i.e., at three, six and 12 months) were considered in analysis set 2 and follow-up interviews between one and three years after ICU discharge (i.e., at 12, 24 and 36 months) in analysis set 3. Note, that patients were excluded from the latter set, if they died or were loss to follow-up prior to follow-up interview 3. All other definitions were as in the analysis set 1. An overview is provided in Supplement 3 Table S4.

**Estimated models.** The models without covariates were estimated for all three analysis sets.

**Reporting.** We report the fitted transition probability matrix at 12 and 36 months after ICU discharge and the probability that the next move of the process is to a specific state. We only consider point estimates and no 95% confidence intervals due to numerical issues in this complex model based on the current data. Furthermore, we provide expected and observed prevalence of the states.

**Software.** For the modelling, we used R (version 4.0.2) and the R package msm.^10^

## Regression analyses

**Regression modelling.** To identify predictors of the ADL score, we performed two multiple linear regression analyses. In the first model, we included all patients who completed the 12-months follow-up interview. In the second model, we included those with completed 36-months follow-up interview. As we did not aim at identifying new predictors or prediction models, we focused on testing reported predictors as identified by a literature search.^11^ Summarizing, the following predictors were tested in the MSC in each of the models: age, sex, maximum SOFA score during ICU treatment, length of ICU stay, number of organ replacement therapies (mechanical ventilation, dialysis, ECMO, other organ replacement therapies), pre-sepsis ADL score, pre-existing renal failure, chronic cardiovascular disease, cancer, chronic cerebrovascular diseases (transient ischemic attack and stroke), liver disease, chronic respiratory disease and dementia, discharge to rehabilitation vs. other discharge. The regression analysis was conducted using the full information maximum likelihood (FIML) technique for handling missing data.

**Reporting.** We report standardized regression coefficients, 95% confidence intervals (CIs) and corresponding p-values.

**Software.** Analyses were conducted using R (version 4.2.3) and the R-package lavaan.^12^

# Supplement: Figures


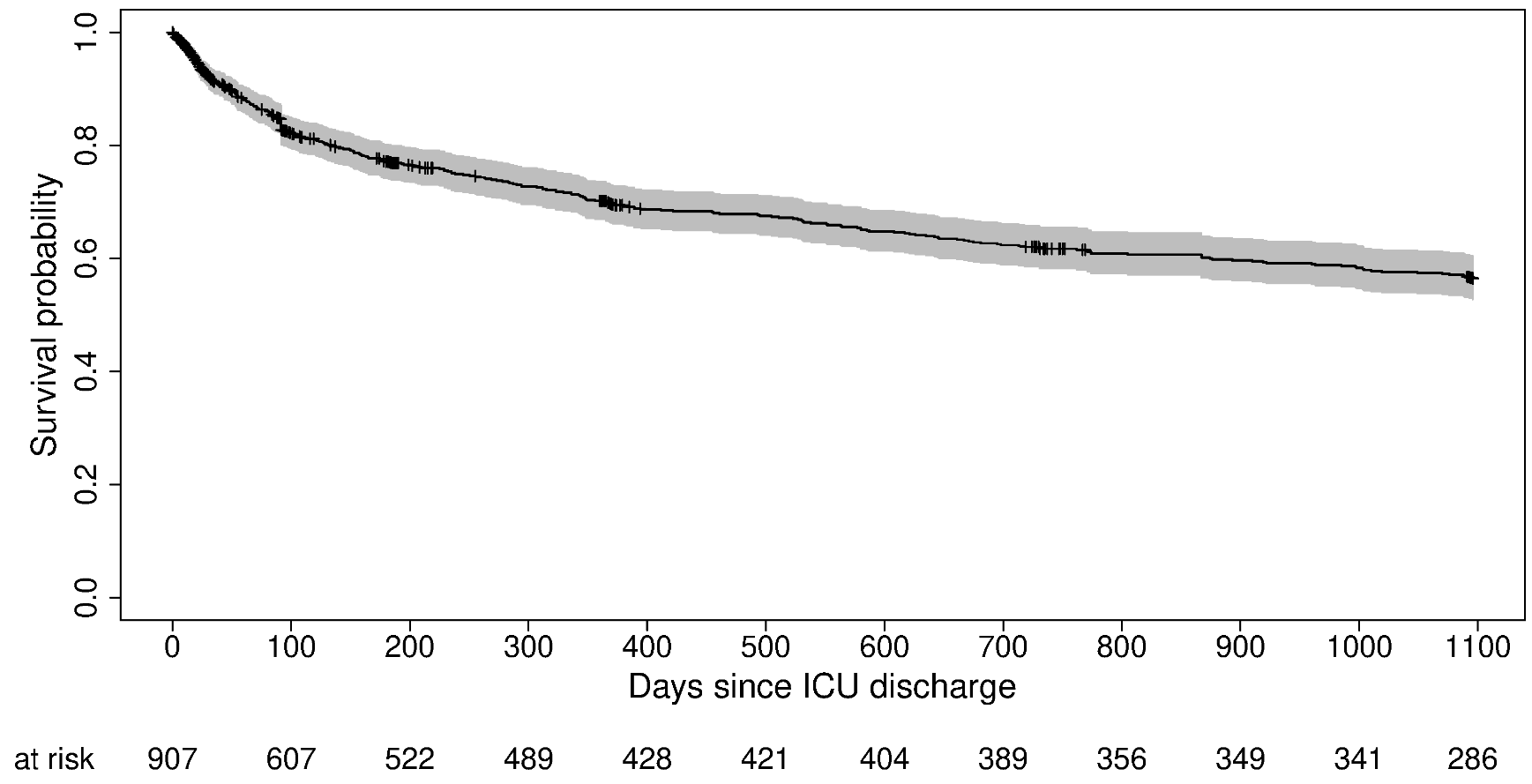


## Figure S1: Kaplan-Meier estimate of overall survival

The 95% confidence intervals of the point estimates are indicated in grey. Censoring is indicated with +. The number of patients at risk is provided below the plot. Note that the step in the curve at three months is introduced by patients, who died between hospital discharge and the first follow-up interview (scheduled at three months after ICU discharge) but for whom a definite date of death was not available. For these patients, the date of death was set to the hypothetical date of the first follow-up interview. Abbreviations: ICU, intensive care unit.


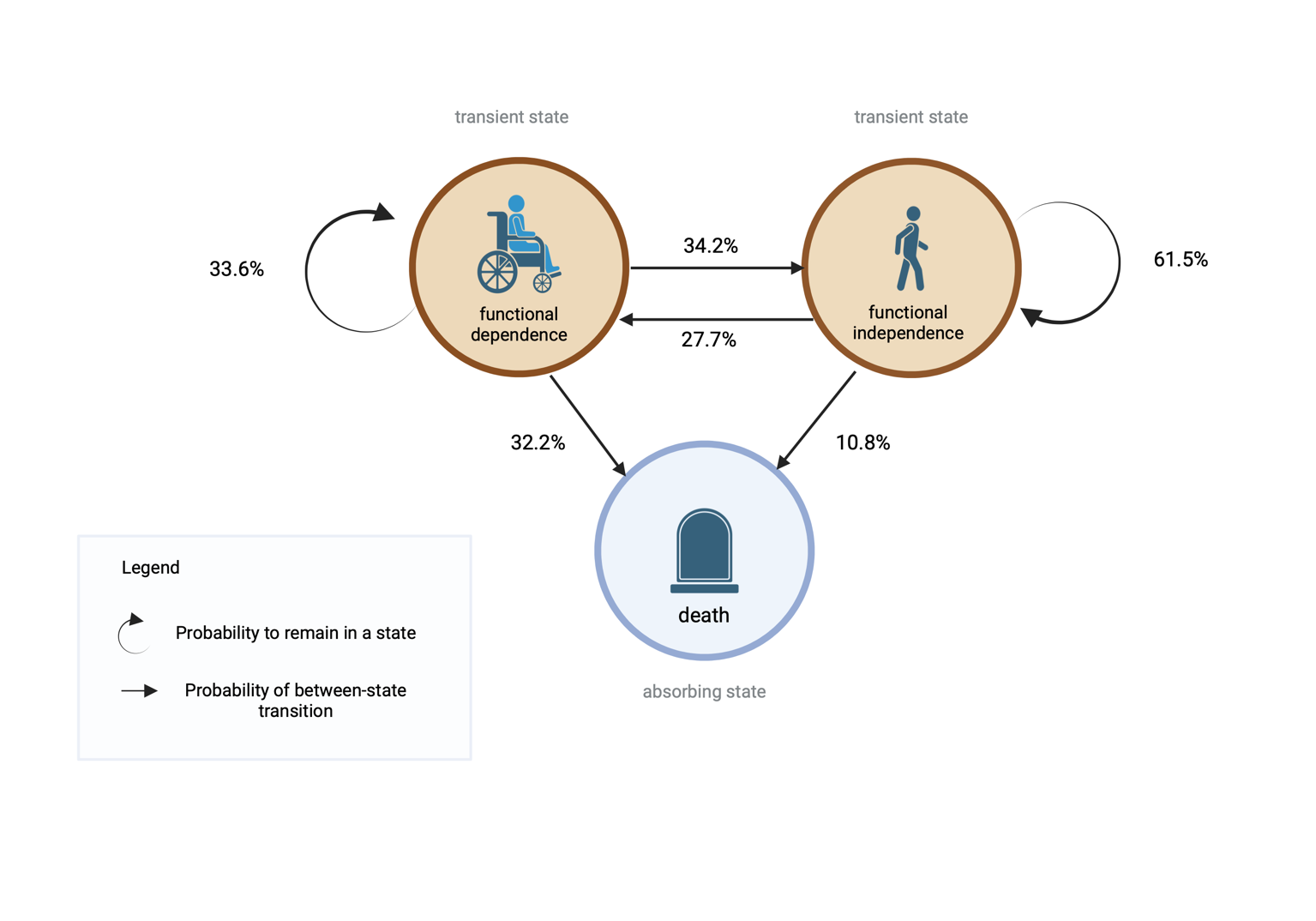


## Figure S2: Transition probabilities within one year after discharge from the intensive care unit (ICU)

Provided are results from the multistate, competing risk modelling for 753 patients (analysis set 2) of the Mid German Sepsis Cohort with available information on their state of dependency during follow-up. Individuals were considered to be in a dependent state at the time of discharge from the ICU. Percentages at the curved arrows indicate the probability to remain in the respective state and percentages at the straight arrows indicate the probability for the respective transition. Figure created with BioRender.com.

**
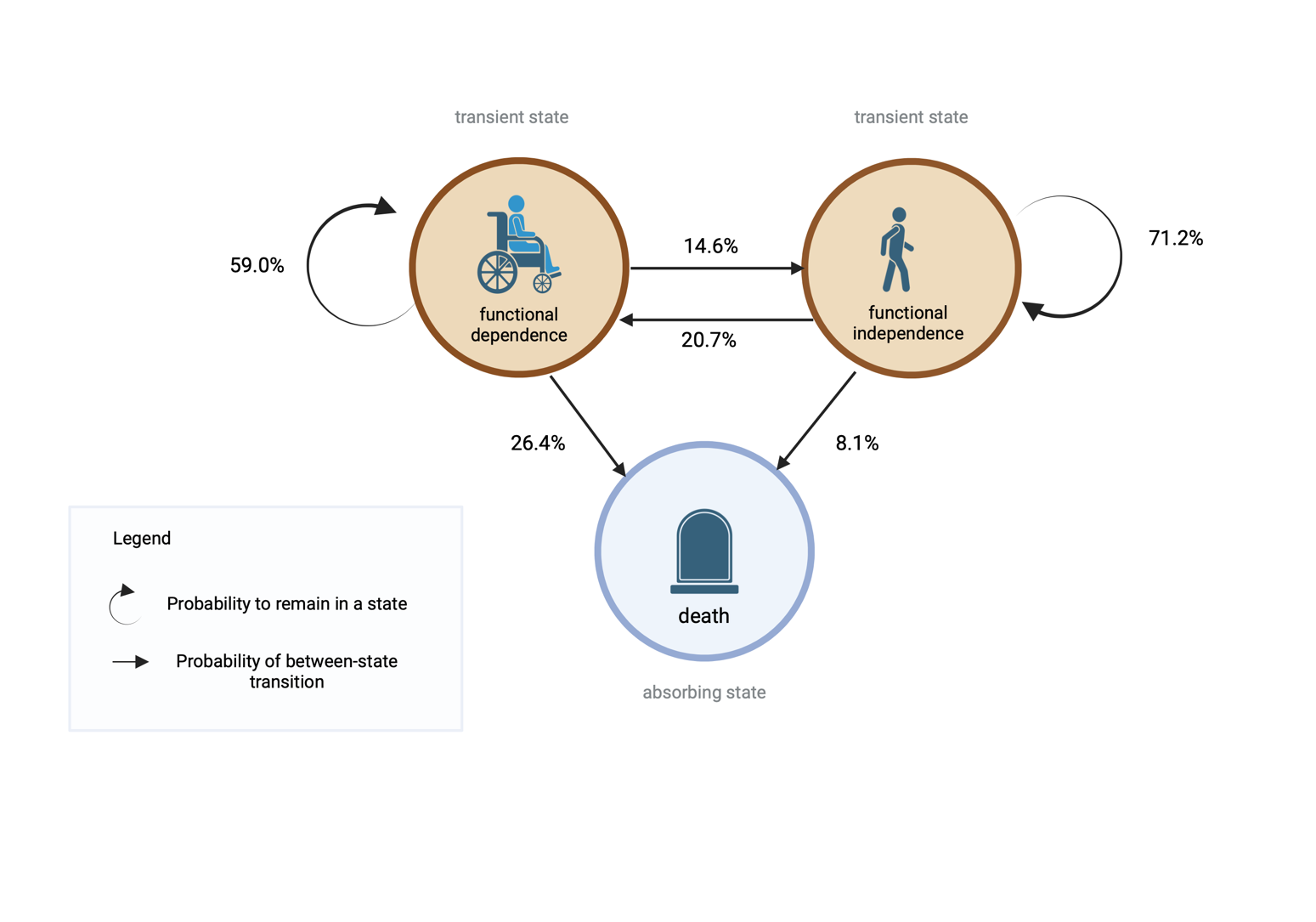
**

## Figure S3: Transition probabilities within one to three years after discharge from the intensive care unit (ICU)

Provided are results from the multistate, competing risk modelling for 429 patients (analysis set 3) of the Mid German Sepsis Cohort with available information on their state of dependency during follow-up. Individuals were considered to be in a dependent state at the time of discharge from the ICU. Percentages at the curved arrows indicate the probability to remain in the respective state and percentages at the straight arrows indicate the probability for the respective transition. Figure created with BioRender.com.


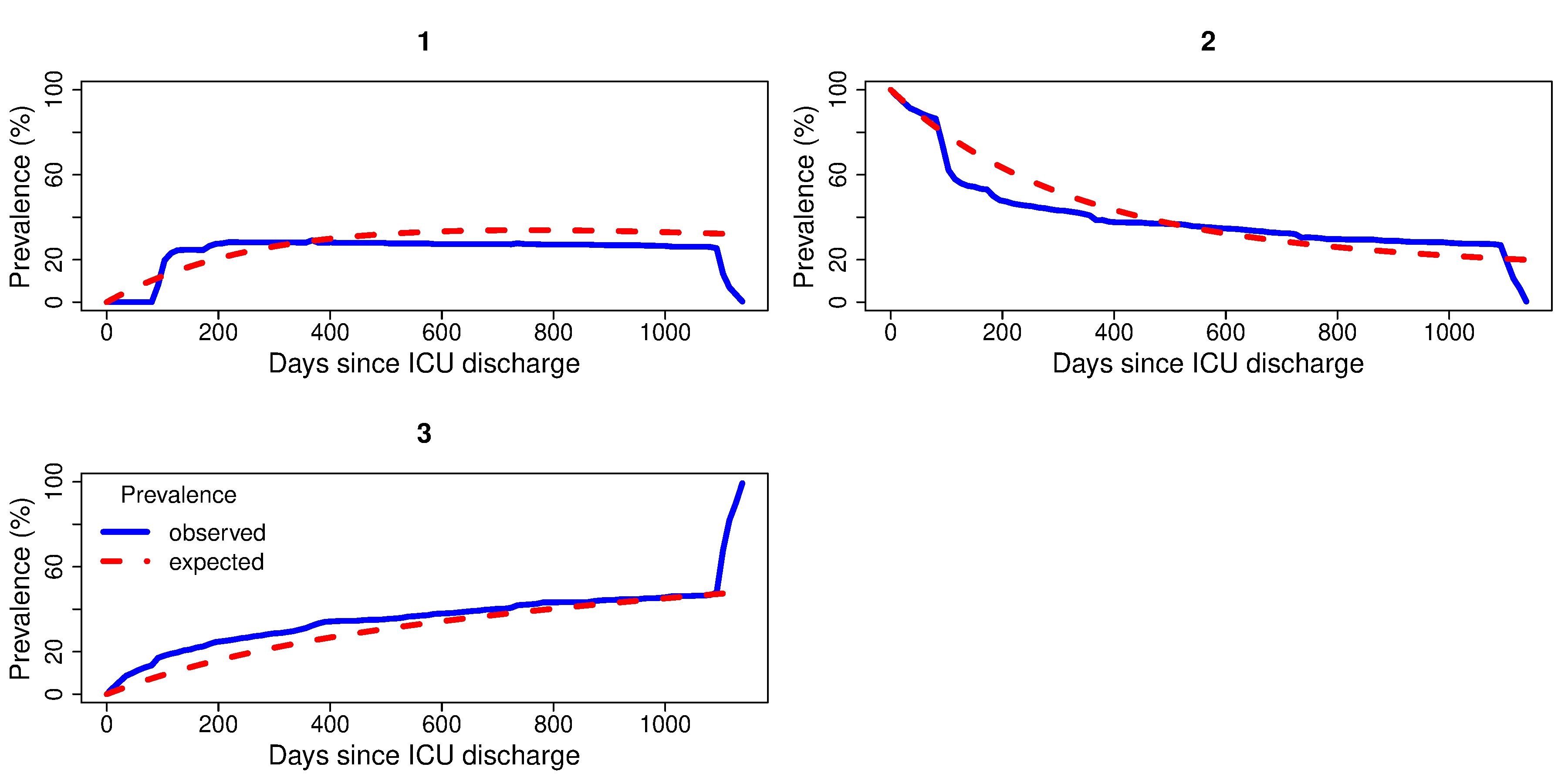


## Figure S4: Observed and expected prevalence of the three states (1: independence, 2: dependence, 3: all-cause death) of the multistate, competing risk model

This model was built based on analysis set 1. The blue solid line describes the observed proportion of patients in the respective state at the given time point in days after discharge from the intensive care unit (ICU). The dashed red line provides the related expected proportion of patients estimated based on the multistate, competing risk model. Note, the deviation between observed and expected values at the extremes of the x-axis is introduced by the design. The first follow-up interview was scheduled at three months after discharge from intensive care unit (ICU) and all patients were dependent (i.e. in state 2) at ICU discharge. The follow-up interviews 2 to 5 were scheduled at six, 12, 24 and 36 months after ICU discharge. Note, the state “dependence” was assigned to patients without state information at ICU discharge.


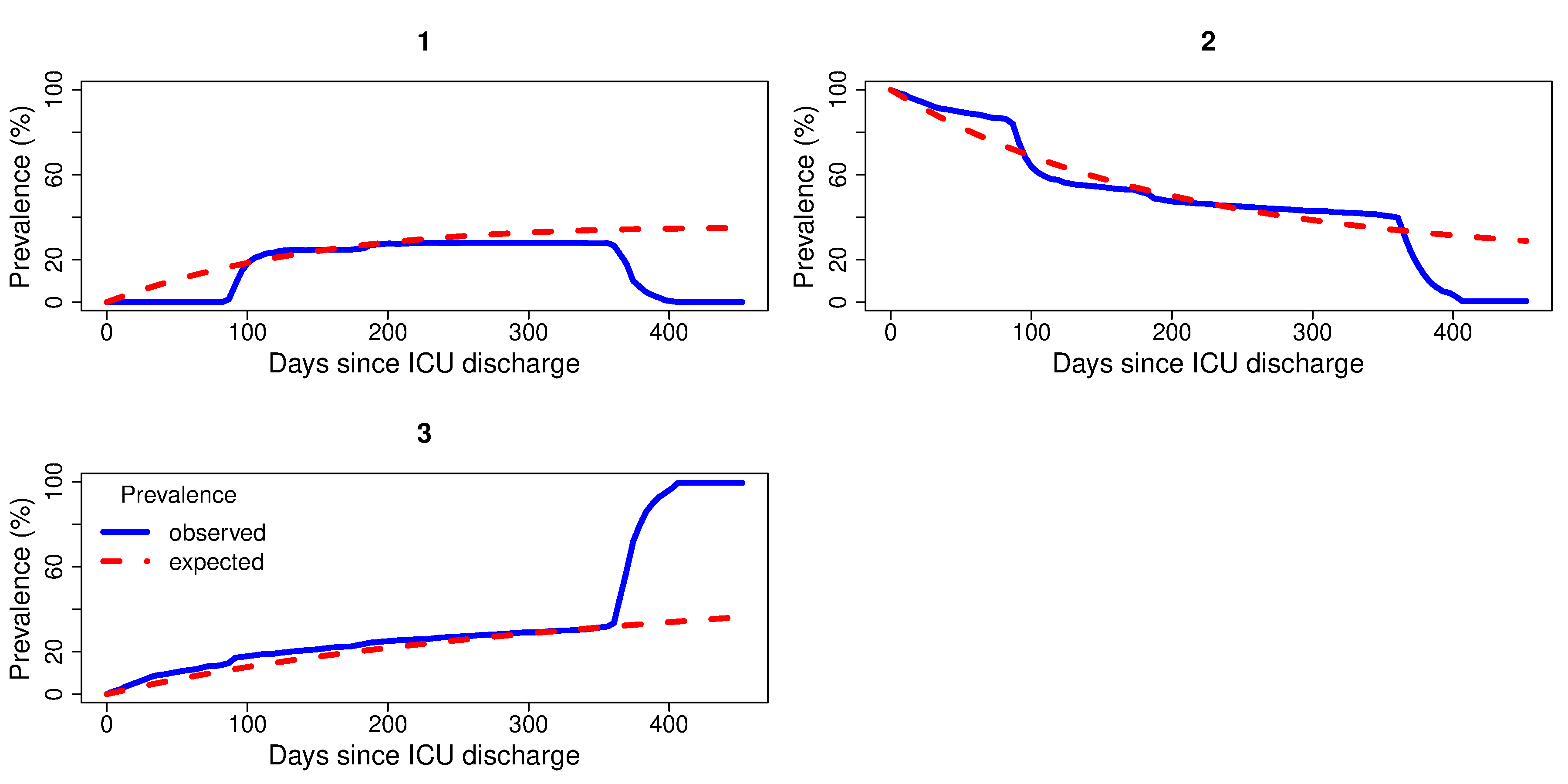


## Figure S5: Observed and expected prevalence of the three states (1: independence, 2: dependence, 3: all-cause death) of the multistate, competing risk model

This model was built based on analysis set 2. The blue solid line describes the observed proportion of patients in the respective state at the given time point in days after discharge from the intensive care unit (ICU). The dashed red line provides the related expected proportion of patients estimated based on the multistate, competing risk model. Note, the deviation between observed and expected values at the extremes of the x-axis is introduced by the design. The first follow-up interview was scheduled at three months after discharge from intensive care unit (ICU) and all patients were dependent (i.e. in state 2) at ICU discharge. The follow-up interviews 2 to 5 were scheduled at six, 12, 24 and 36 months after ICU discharge. Note, the state “dependence” was assigned to patients without state information at ICU discharge.


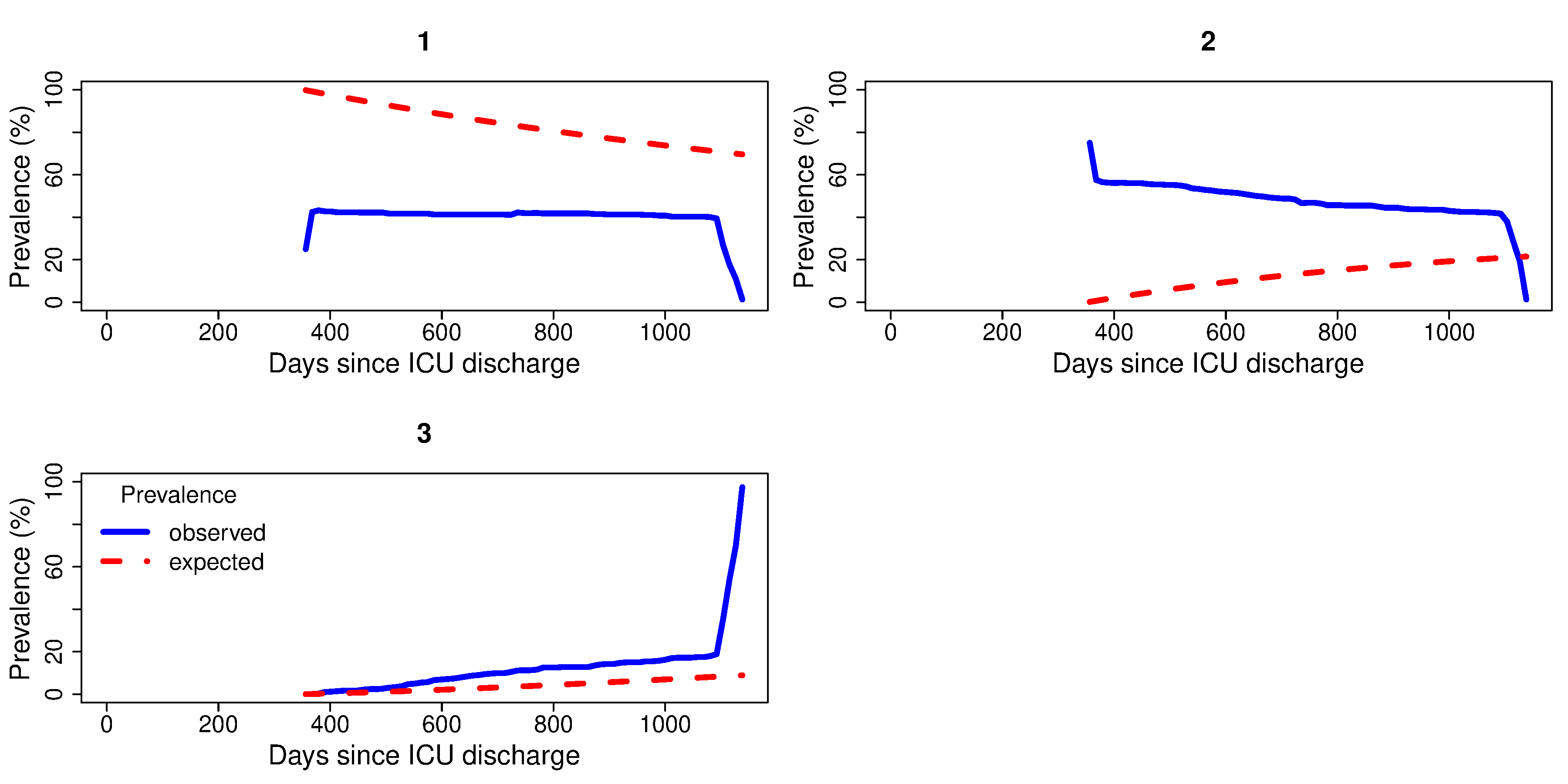


## Figure S6: Observed and expected prevalence of the three states (1: independence, 2: dependence, 3: all-cause death) of the multistate, competing risk model

This model was built based on analysis set 3. The blue solid line describes the observed proportion of patients in the respective state at the given time point in days after discharge from the intensive care unit (ICU). The dashed red line provides the related expected proportion of patients estimated based on the multistate, competing risk model. Note, the deviation between observed and expected values at the extremes of the x-axis is introduced by the design. The first follow-up interview was scheduled at three months after discharge from intensive care unit (ICU) and all patients were dependent (i.e. in state 2) at ICU discharge. The follow-up interviews 2 to 5 were scheduled at six, 12, 24 and 36 months after ICU discharge. However, the concordance between the observed and expected prevalence is, at least for state 1 and 2, questionable. Note, the state “dependence” was assigned to patients without state information at ICU discharge.


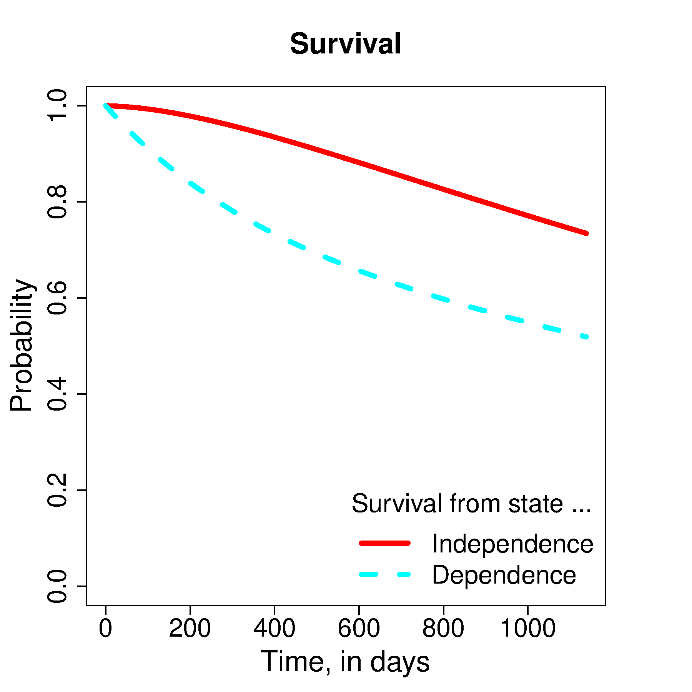

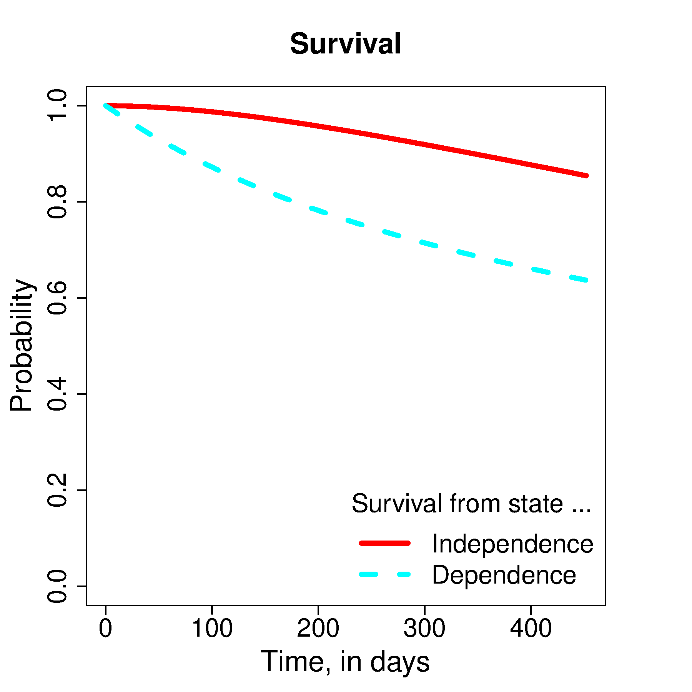

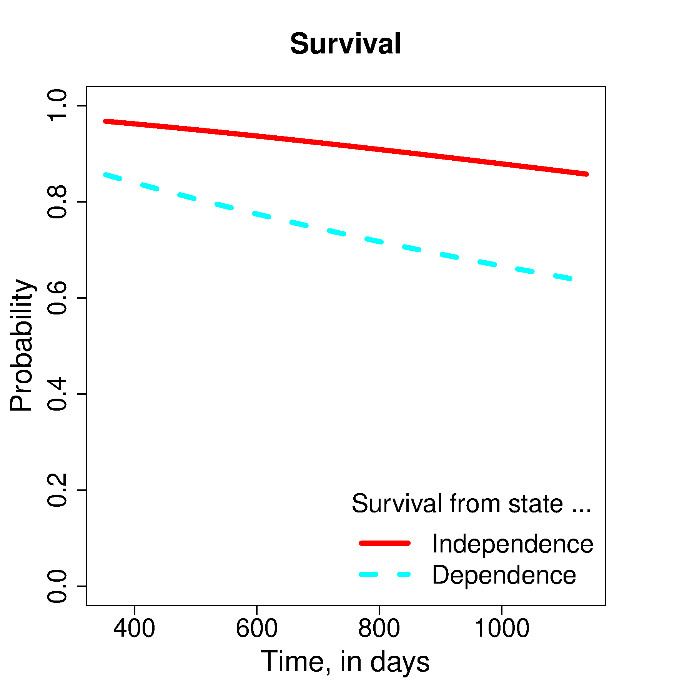


## Figure S7: Fitted survival from the two transient states (independence, dependence) of the multistate, competing risk models

These models were built in analysis set 1 (left panel), analysis set 2 (middle panel) and analysis set 3 (right panel). The probability to be alive at a specific time point in days since discharge from the intensive care unit (ICU) is larger for patients being independent (red solid line) compared to those being dependent (cyan dashed line). Note, the state “dependence” was assigned to patients without state information at discharge from ICU.

# Supplement: Tables

## Table S1: Overview of included data for multistate, competing risk modelling and the related time points after intensive care unit (ICU) discharge, for which the transition probabilities are estimated

|  | Analysis set 1 | Analysis set 2 | Analysis set 3 |
| --- | --- | --- | --- |
| Number of patients | 753 | 753 | 429 |
| Data for model estimation |  |  |  |
| baseline (hospital stay) | x | x | - |
| follow-up interview at … |  |  |  |
| … 3 months after ICU discharge | x | x | - |
| … 6 months after ICU discharge | x | x | - |
| … 12 months after ICU discharge | x | x | x |
| … 24 months after ICU discharge | x | - | x |
| … 36 months after ICU discharge | x | - | x |
| Transition probability estimation for … |  |  |  |
| … 12 months after ICU discharge | x | x | - |
| … 36 months after ICU discharge | x | - | x |

Baseline comprise information gained during the hospital stay. Of note, only patients, who were still alive and an active member of the MSC at 12 months, could be included in the analysis set 3. Cross indicates applicability and hyphen the non-applicability for the respective model.

## Table S2: Interview characteristics

| Characteristic | FU 1  (N = 590) | FU 2  (N = 537) | FU 3  (N = 459) | FU 4  (N = 375) | FU 5  (N = 330) |
| --- | --- | --- | --- | --- | --- |
| Interviewed person |  |  |  |  |  |
| patient; n (%) | 340 (57.6%) | 374 (69.6%) | 337 (73.4%) | 296 (78.9%) | 268 (81.2%) |
| proxy; n (%) | 156 (26.4%) | 100 (18.6%) | 78 (17.0%) | 44 (11.7%) | 36 (10.9%) |
| both*; n (%) | 94 (15.9%) | 63 (11.7%) | 44 (9.6%) | 35 (9.3%) | 26 (7.9%) |
| Type of interview |  |  |  |  |  |
| by telephone; n (%) | 561 (95.1%) | 523 (97.4%) | 451 (98.3%) | 371 (98.9%) | 328 (99.4%) |
| in person; n (%) | 29 (4.9%) | 14 (2.6%) | 8 (1.7%) | 4 (1.1%) | 2 (0.6%) |
| Days since ICU discharge |  |  |  |  |  |
| median (Q1, Q3) | 94 (91, 101) | 185 (182, 191) | 366 (363, 372) | 731 (728, 736) | 1101 (1097, 1113) |
| Duration of interview, in hours |  |  |  |  |  |
| median (Q1, Q3) | 1.0 (1.0, 1.3) | 0.8 (0.8, 1.0) | 0.9 (0.8, 1.0) | 0.8 (0.8, 1.0) | 0.8 (0.8, 1.0) |

Absolute and relative frequencies (n, %) or median with first and third quartile (Q1, Q3) are provided. Furthermore, the number of patients who completed the respective FU interview is given (N). Abbreviations: FU, follow-up; ICU, intensive care unit. *In case of disagreement between patient and proxy, the response of the patient was used.

## Table S3: Impairments post-sepsis identified by validated questionnaires for all time points of MSC follow-up (FU) assessments (longitudinal perspective)

|  | **Instrument** |  |  | **Longitudinal (N = 330)** | | | | |
| --- | --- | --- | --- | --- | --- | --- | --- | --- |
|  |  |  |  | | **n_ass_** | **n_valid_** | **n** | **% [95% CI]** |
| **3 months** | T-MoCA |  |  | | 216 | 215 | 44 | 20.5 [15.6, 26.4] |
|  | IQCODE |  |  | | 98 | 65 | 37 | 56.9 [44.8, 68.2] |
|  | Cognitive |  |  | | 310 | 278 | 80 | 28.8 [23.8, 34.4] |
|  | BSI-18 |  |  | | 318 | 275 | 42 | 15.3 [11.5, 20.0] |
|  | PTSS-10 |  |  | | 317 | 302 | 36 | 11.9 [8.7, 16.1] |
|  | Psychological |  |  | | 319 | 274 | 56 | 20.4 [16.1, 25.6] |
|  | CFS |  |  | | 321 | 313 | 211 | 67.4 [62, 72.4] |
| **6 months** | T-MoCA |  |  | | 238 | 235 | 46 | 19.6 [15.0, 25.1] |
|  | IQCODE |  |  | | 68 | 48 | 29 | 60.4 [46.3, 73.0] |
|  | Cognitive |  |  | | 306 | 283 | 75 | 26.5 [21.7, 31.9] |
|  | BSI-18 |  |  | | 322 | 292 | 35 | 12.0 [8.7, 16.2] |
|  | PTSS-10 |  |  | | 322 | 315 | 38 | 12.1 [8.9, 16.1] |
|  | Psychological |  |  | | 323 | 292 | 49 | 16.8 [12.9, 21.5] |
|  | CFS |  |  | | 323 | 315 | 207 | 65.7 [60.3, 70.7] |
| **1 year** | T-MoCA |  |  | | 251 | 249 | 38 | 15.3 [11.3, 20.3] |
|  | IQCODE |  |  | | 59 | 48 | 33 | 68.8 [54.7, 80.1] |
|  | Cognitive |  |  | | 310 | 297 | 71 | 23.9 [19.4, 29.1] |
|  | BSI-18 |  |  | | 323 | 286 | 35 | 12.2 [8.9, 16.5] |
|  | PTSS-10 |  |  | | 322 | 318 | 39 | 12.3 [9.1, 16.3] |
|  | Psychological |  |  | | 324 | 290 | 48 | 16.6 [12.7, 21.3] |
|  | CFS |  |  | | 325 | 319 | 221 | 69.3 [64, 74.1] |
| **2 years** | T-MoCA |  |  | | 237 | 237 | 34 | 14.3 [10.5, 19.4] |
|  | IQCODE |  |  | | 47 | 32 | 26 | 81.2 [64.7, 91.1] |
|  | Cognitive |  |  | | 284 | 269 | 60 | 22.3 [17.7, 27.6] |
|  | BSI-18 |  |  | | 322 | 281 | 34 | 12.1 [8.8, 16.4] |
|  | PTSS-10 |  |  | | 321 | 314 | 32 | 10.2 [7.3, 14.0] |
|  | Psychological |  |  | | 322 | 284 | 49 | 17.3 [13.3, 22.1] |
|  | CFS |  |  | | 323 | 320 | 230 | 71.9 [66.7, 76.5] |
| **3 years** | T-MoCA |  |  | | 207 | 206 | 26 | 12.6 [8.8, 17.9] |
|  | IQCODE |  |  | | 60 | 41 | 27 | 65.9 [50.5, 78.4] |
|  | Cognitive |  |  | | 267 | 247 | 53 | 21.5 [16.8, 27.0] |
|  | BSI-18 |  |  | | 327 | 284 | 29 | 10.2 [7.2, 14.3] |
|  | PTSS-10 |  |  | | 326 | 321 | 32 | 10.0 [7.2, 13.7] |
|  | Psychological |  |  | | 328 | 287 | 44 | 15.3 [11.6, 20.0] |
|  | CFS |  |  | | 329 | 327 | 248 | 75.8 [70.9, 80.2] |

N = number of interviewed patients. n_ass_ = number of patients assessed by the questionnaire. n_valid_ = number of valid assessments. n = number of patients with an impairment identified in the assessment. % = proportion of patients with an impairment in the assessment. Cognitive = assessment with T-MoCA or IQCODE. Psychological = assessment with BSI-18 or PTSS-10. CFS = Chalder Fatigue Scale.

## Table S4: Prevalent symptoms reported by interviewees for all time points of MSC follow-up assessments – cross-sectional perspective

| **Time post-sepsis** |  | **3 months** | |  | **6 months** | |  | **1 year** | |  | **2 years** | |  | **3 years** | |
| --- | --- | --- | --- | --- | --- | --- | --- | --- | --- | --- | --- | --- | --- | --- | --- |
| **Patients** |  | **N = 590** | |  | **N = 537** | |  | **N = 459** | |  | **N = 375** | |  | **N = 330** | |
| **Impairment** |  | **n** | **% [95%-CI]** |  | **n** | **% [95%-CI]** |  | **n** | **% [95%-CI]** |  | **n** | **% [95%-CI]** |  | **n** | **% [95%-CI]** |
| Cognitive |  | 286 | 48.5 [44.5, 52.5] |  | 272 | 50.7 [46.4, 54.9] |  | 264 | 57.5 [53.0, 62.0] |  | 226 | 60.3 [55.2, 65.1] |  | 208 | 63.0 [57.7, 68.1] |
| Psychological |  | 275 | 46.6 [42.6, 50.6] |  | 244 | 45.4 [41.3, 49.7] |  | 226 | 49.2 [44.7, 53.8] |  | 195 | 52.0 [46.9, 57.0] |  | 150 | 45.5 [40.2, 50.8] |
| Physical |  | 563 | 95.4 [93.4, 96.8] |  | 500 | 93.1 [90.6, 95.0] |  | 421 | 91.7 [88.8, 93.9] |  | 345 | 92.0 [88.8, 94.3] |  | 304 | 92.1 [88.7, 94.6] |
| Sensoric |  | 242 | 41.0 [37.1, 45.0] |  | 199 | 37.1 [33.1, 41.2] |  | 172 | 37.5 [33.2, 42.0] |  | 151 | 40.3 [35.4, 45.3] |  | 145 | 43.9 [38.7, 49.3] |
| Renal |  | 58 | 9.8 [7.7, 12.5] |  | 44 | 8.2 [6.2, 10.8] |  | 39 | 8.5 [6.3, 11.4] |  | 24 | 6.4 [4.3, 9.3] |  | 19 | 5.8 [3.7, 8.8] |
| Respiratory |  | 255 | 43.2 [39.3, 47.2] |  | 234 | 43.6 [39.4, 47.8] |  | 207 | 45.1 [40.6, 49.7] |  | 184 | 49.1 [44.0, 54.1] |  | 168 | 50.9 [45.5, 56.3] |
| Dysphagic |  | 93 | 15.8 [13.0, 18.9] |  | 69 | 12.8 [10.3, 15.9] |  | 69 | 15.0 [12.1, 18.6] |  | 73 | 19.5 [15.8, 23.8] |  | 60 | 18.2 [14.4, 22.7] |
| Muscular |  | 409 | 69.3 [65.5, 72.9] |  | 322 | 60.0 [55.8, 64.0] |  | 261 | 56.9 [52.3, 61.3] |  | 191 | 50.9 [45.9, 56.0] |  | 210 | 63.6 [58.3, 68.6] |
| Walk |  | 303 | 51.4 [47.3, 55.4] |  | 251 | 46.7 [42.6, 51.0] |  | 211 | 46.0 [41.5, 50.5] |  | 174 | 46.4 [41.4, 51.5] |  | 171 | 51.8 [46.4, 57.2] |
| Mis- or malsensation |  | 273 | 46.3 [42.3, 50.3] |  | 259 | 48.2 [44.0, 52.5] |  | 251 | 54.7 [50.1, 59.2] |  | 202 | 53.9 [48.8, 58.8] |  | 190 | 57.6 [52.2, 62.8] |
| Weight |  | 132 | 22.4 [19.2, 25.9] |  | 69 | 12.8 [10.3, 15.9] |  | 34 | 7.4 [5.3, 10.2] |  | 14 | 3.7 [2.2, 6.2] |  | 11 | 3.3 [1.9, 5.9] |
| Incontinence |  | 115 | 19.5 [16.5, 22.9] |  | 95 | 17.7 [14.7, 21.1] |  | 81 | 17.6 [14.4, 21.4] |  | 64 | 17.1 [13.6, 21.2] |  | 62 | 18.8 [14.9, 23.4] |
| Sexual |  | 120 | 20.3 [17.3, 23.8] |  | 115 | 21.4 [18.2, 25.1] |  | 114 | 24.8 [21.1, 29.0] |  | 104 | 27.7 [23.4, 32.5] |  | 99 | 30.0 [25.3, 35.2] |
| Pain |  | 263 | 44.6 [40.6, 48.6] |  | 242 | 45.1 [40.9, 49.3] |  | 201 | 43.8 [39.3, 48.4] |  | 174 | 46.4 [41.4, 51.5] |  | 166 | 43.9 [38.7, 49.3] |
| Other |  | 362 | 61.4 [57.4, 65.2] |  | 323 | 60.1 [56.0, 64.2] |  | 276 | 60.1 [55.6, 64.5] |  | 228 | 60.8 [55.8, 65.6] |  | 203 | 61.5 [56.2, 66.6] |
| Fatigue |  | 197 | 33.4 [29.7, 37.3] |  | 190 | 35.4 [31.5, 39.5] |  | 174 | 37.9 [33.6, 42.4] |  | 148 | 39.5 [34.7, 44.5] |  | 148 | 44.8 [39.6, 50.2] |
| Sleep |  | 287 | 48.6 [44.6, 52.7] |  | 239 | 44.5 [40.4, 48.7] |  | 197 | 42.9 [38.5, 47.5] |  | 163 | 43.5 [38.5, 48.5] |  | 138 | 41.8 [36.6, 47.2] |

N = number of patients included in the assessment; n = number of patients reporting the respective impairment; % = proportion of patients reporting the respective impairment; CI = confidence interval.

## Table S5: New onset symptoms reported by interviewees for all time points of MSC follow-up assessments – cross-sectional perspective

| **Time post-sepsis** |  | **3 months** | |  | **6 months** | |  | **1 year** | |  | **2 years** | |  | **3 years** | |
| --- | --- | --- | --- | --- | --- | --- | --- | --- | --- | --- | --- | --- | --- | --- | --- |
| **Patients** |  | **N = 590** | |  | **N = 537** | |  | **N = 459** | |  | **N = 375** | |  | **N = 330** | |
| **Impairment** |  | **n** | **% [95%-CI]** |  | **n** | **% [95%-CI]** |  | **n** | **% [95%-CI]** |  | **n** | **% [95%-CI]** |  | **n** | **% [95%-CI]** |
| Cognitive |  | 216 | 36.6 [32.8, 40.6] |  | 209 | 38.9 [34.9, 43.1] |  | 218 | 47.5 [43.0, 52.1] |  | 202 | 53.9 [48.8, 58.8] |  | 191 | 57.9 [52.5, 63.1] |
| Psychological |  | 206 | 34.9 [31.2, 38.8] |  | 196 | 36.5 [32.5, 40.7] |  | 203 | 44.2 [39.7, 48.8] |  | 172 | 45.9 [40.9, 50.9] |  | 135 | 40.9 [35.7, 46.3] |
| Physical |  | 530 | 89.8 [87.1, 92.0] |  | 474 | 88.3 [85.3, 90.7] |  | 408 | 88.9 [85.7, 91.4] |  | 340 | 90.7 [87.3, 93.2] |  | 301 | 91.2 [87.7, 93.8] |
| Sensoric |  | 116 | 19.7 [16.7, 23.1] |  | 91 | 16.9 [14.0, 20.4] |  | 95 | 20.7 [17.2, 24.6] |  | 86 | 22.9 [19.0, 27.5] |  | 90 | 27.3 [22.7, 32.3] |
| Renal |  | 29 | 4.9 [3.4, 7.0] |  | 25 | 4.7 [3.2, 6.8] |  | 19 | 4.1 [2.7, 6.4] |  | 13 | 3.5 [2.0, 5.8] |  | 12 | 3.6 [2.1, 6.2] |
| Respiratory |  | 105 | 17.8 [14.9, 21.1] |  | 126 | 23.5 [20.1, 27.2] |  | 107 | 23.3 [19.7, 27.4] |  | 116 | 30.9 [26.5, 35.8] |  | 119 | 36.1 [31.1, 41.4] |
| Dysphagic |  | 67 | 11.4 [ 9.0, 14.2] |  | 52 | 9.7 [7.5, 12.5] |  | 58 | 12.6 [9.9, 16.0] |  | 59 | 15.7 [12.4, 19.8] |  | 48 | 14.5 [11.1, 18.8] |
| Muscular |  | 333 | 56.4 [52.4, 60.4] |  | 267 | 49.7 [45.5, 53.9] |  | 230 | 50.1 [45.6, 54.7] |  | 172 | 45.9 [40.9, 50.9] |  | 193 | 58.5 [53.1, 63.7] |
| Walk |  | 210 | 35.6 [31.8, 39.5] |  | 178 | 33.1 [29.3, 37.2] |  | 164 | 35.7 [31.5, 40.2] |  | 143 | 38.1 [33.4, 43.1] |  | 153 | 46.4 [41.1, 51.8] |
| Mis- or malsensation |  | 158 | 26.8 [23.4, 30.5] |  | 162 | 30.2 [26.4, 34.2] |  | 179 | 39.0 [34.6, 43.5] |  | 150 | 40.0 [35.2, 45.0] |  | 146 | 44.2 [39.0, 49.6] |
| Weight |  | 107 | 18.1 [15.2, 21.4] |  | 60 | 11.2 [8.8, 14.1] |  | 32 | 7.0 [5.0, 9.7] |  | 14 | 3.7 [2.2, 6.2] |  | 10 | 3.0 [1.7, 5.5] |
| Incontinence |  | 72 | 12.2 [ 9.8, 15.1] |  | 69 | 12.8 [10.3, 15.9] |  | 63 | 13.7 [10.9, 17.2] |  | 53 | 14.1 [11.0, 18.0] |  | 56 | 17.0 [13.3, 21.4] |
| Sexual |  | 47 | 8.0 [6.0, 10.4] |  | 62 | 11.5 [9.1, 14.5] |  | 69 | 15.0 [12.1, 18.6] |  | 80 | 21.3 [17.5, 25.8] |  | 83 | 25.2 [20.8, 30.1] |
| Pain |  | 121 | 20.5 [17.4, 24.0] |  | 127 | 23.6 [20.3, 27.4] |  | 113 | 24.6 [20.9, 28.8] |  | 116 | 30.9 [26.5, 35.8] |  | 118 | 35.8 [30.8, 41.1] |
| Other |  | 218 | 36.9 [33.2, 40.9] |  | 224 | 41.7 [37.6, 45.9] |  | 211 | 46.0 [41.5, 50.5] |  | 178 | 47.5 [42.5, 52.5] |  | 161 | 48.8 [43.4, 54.2] |
| Fatigue |  | 121 | 20.5 [17.4, 24.0] |  | 141 | 26.3 [22.7, 30.1] |  | 143 | 31.2 [27.1, 35.5] |  | 124 | 33.1 [28.5, 38.0] |  | 122 | 37.0 [31.9, 42.3] |
| Sleep |  | 131 | 22.2 [19.0, 25.7] |  | 129 | 24.0 [20.6, 27.8] |  | 118 | 25.7 [21.9, 29.9] |  | 97 | 25.9 [21.7, 30.5] |  | 85 | 25.8 [21.3, 30.7] |

N = number of patients included in the assessment; n = number of patients reporting the respective impairment; % = proportion of patients reporting the respective impairment; CI = confidence interval.

## Table S6: Prevalent symptoms reported by three-year follow-up patients (N = 330) for all time points of MSC follow-up assessments – longitudinal perspective

| **Time post-sepsis** |  | **3 months** | |  | **6 months** | |  | **1 year** | |  | **2 years** | |  | **3 years** | |
| --- | --- | --- | --- | --- | --- | --- | --- | --- | --- | --- | --- | --- | --- | --- | --- |
| **Patients** |  | **N = 321** | |  | **N = 324** | |  | **N = 325** | |  | **N = 324** | |  | **N = 330** | |
| **Impairment** |  | **n** | **% [95%-CI]** |  | **n** | **% [95%-CI]** |  | **n** | **% [95%-CI]** |  | **n** | **% [95%-CI]** |  | **n** | **% [95%-CI]** |
| Cognitive |  | 149 | 46.4 [41.0, 51.9] |  | 163 | 50.3 [44.9, 55.7] |  | 183 | 56.3 [50.9, 61.6] |  | 195 | 59.4 [54.0, 64.6] |  | 208 | 63.0 [57.7, 68.1] |
| Psychological |  | 143 | 44.5 [39.2, 50.0] |  | 146 | 45.1 [39.7, 50.5] |  | 159 | 48.9 [43.5, 54.3] |  | 169 | 52.2 [46.7, 57.5] |  | 150 | 45.5 [40.2, 50.8] |
| Physical |  | 305 | 95 [92.1, 96.9] |  | 302 | 93.2 [89.9, 95.5] |  | 298 | 91.7 [88.2, 94.2] |  | 297 | 91.7 [88.1, 94.2] |  | 304 | 92.1 [88.7, 94.6] |
| Sensoric |  | 135 | 42.1 [36.8, 47.5] |  | 121 | 37.3 [32.3, 42.7] |  | 120 | 36.9 [31.9, 42.3] |  | 130 | 60.2 [54.8, 65.4] |  | 145 | 43.9 [38.7, 49.3] |
| Renal |  | 23 | 7.2 [4.8, 10.5] |  | 19 | 5.9 [3.8, 9.0] |  | 23 | 7.1 [4.8, 10.4] |  | 21 | 40.1 [34.9, 45.5] |  | 19 | 5.8 [3.7, 8.8] |
| Respiratory |  | 125 | 38.9 [33.8, 44.4] |  | 140 | 43.2 [37.9, 48.7] |  | 143 | 44.0 [38.7, 49.4] |  | 155 | 6.5 [4.3, 9.7] |  | 168 | 50.9 [45.5, 56.3] |
| Dysphagic |  | 44 | 13.7 [10.4, 17.9] |  | 39 | 12.0 [8.9, 16.0] |  | 48 | 14.8 [11.3, 19.0] |  | 58 | 47.8 [42.5, 53.3] |  | 60 | 18.2 [14.4, 22.7] |
| Muscular |  | 213 | 66.4 [61.0, 71.3] |  | 180 | 55.6 [50.1, 60.9] |  | 178 | 54.8 [49.3, 60.1] |  | 165 | 17.9 [14.1, 22.4] |  | 210 | 63.6 [58.3, 68.6] |
| Walk |  | 152 | 47.4 [42.0, 52.8] |  | 136 | 42.0 [36.7, 47.4] |  | 142 | 43.7 [38.4, 49.1] |  | 144 | 50.9 [45.5, 56.3] |  | 171 | 51.8 [46.4, 57.2] |
| Mis- or malsensation |  | 156 | 48.6 [43.2, 54.0] |  | 160 | 49.4 [44.0, 54.8] |  | 180 | 55.4 [49.9, 60.7] |  | 177 | 44.4 [39.1, 49.9] |  | 190 | 57.6 [52.2, 62.8] |
| Weight |  | 64 | 19.9 [15.9, 24.7] |  | 41 | 12.7 [9.5, 16.7] |  | 24 | 7.4 [5.0, 10.8] |  | 10 | 54.6 [49.2, 60.0] |  | 11 | 3.3 [1.9, 5.9] |
| Incontinence |  | 57 | 17.8 [14.0, 22.3] |  | 58 | 17.9 [14.1, 22.4] |  | 54 | 16.6 [13.0, 21.0] |  | 56 | 3.1 [1.7, 5.6] |  | 62 | 18.8 [14.9, 23.4] |
| Sexual |  | 68 | 21.2 [17.1, 26.0] |  | 75 | 23.1 [18.9, 28.0] |  | 91 | 28.0 [23.4, 33.1] |  | 97 | 17.3 [13.6, 21.8] |  | 99 | 30.0 [25.3, 35.2] |
| Pain |  | 141 | 43.9 [38.6, 49.4] |  | 150 | 46.3 [40.9, 51.7] |  | 143 | 44.0 [38.7, 49.4] |  | 149 | 29.9 [25.2, 35.1] |  | 166 | 50.3 [44.9, 55.7] |
| Other |  | 193 | 60.1 [54.7, 65.3] |  | 188 | 58.0 [52.6, 63.3] |  | 193 | 59.4 [54.0, 64.6] |  | 195 | 60.2 [54.8, 65.4] |  | 203 | 61.5 [56.2, 66.6] |
| Fatigue |  | 96 | 29.9 [25.2, 35.1] |  | 116 | 35.8 [30.8, 41.2] |  | 126 | 38.8 [33.6, 44.2] |  | 126 | 38.9 [33.7, 44.3] |  | 148 | 44.8 [39.6, 50.2] |
| Sleep |  | 151 | 47.0 [41.6, 52.5] |  | 141 | 43.5 [38.2, 49.0] |  | 136 | 41.8 [36.6, 47.3] |  | 142 | 43.8 [38.5, 49.3] |  | 138 | 41.8 [36.6, 47.2] |

N = number of patients included in the assessment; n = number of patients reporting the respective impairment; % = proportion of patients reporting the respective impairment; CI = confidence interval.

## Table S7: New onset symptoms reported by three-year follow-up patients (N = 330) for all time points of MSC follow-up assessments – longitudinal perspective

| **Time post-sepsis** |  | **3 months** | |  | **6 months** | |  | **1 year** | |  | **2 years** | |  | **3 years** | |
| --- | --- | --- | --- | --- | --- | --- | --- | --- | --- | --- | --- | --- | --- | --- | --- |
| **Patients** |  | **N = 321** | |  | **N = 324** | |  | **N = 325** | |  | **N = 324** | |  | **N = 330** | |
| **Impairment** |  | **n** | **% [95%-CI]** |  | **n** | **% [95%-CI]** |  | **n** | **% [95%-CI]** |  | **n** | **% [95%-CI]** |  | **n** | **% [95%-CI]** |
| Cognitive |  | 118 | 36.8 [31.7, 42.2] |  | 134 | 41.4 [36.1, 46.8] |  | 154 | 47.4 [42.0, 52.8] |  | 177 | 54.6 [49.2, 60.0] |  | 191 | 57.9 [52.5, 63.1] |
| Psychological |  | 114 | 35.5 [30.5, 40.9] |  | 120 | 37.0 [32.0, 42.4] |  | 149 | 45.8 [40.5, 51.3] |  | 153 | 47.2 [41.9, 52.7] |  | 135 | 40.9 [35.7, 46.3] |
| Physical |  | 289 | 90.0 [86.3, 92.8] |  | 287 | 88.6 [84.7, 91.6] |  | 289 | 88.9 [85.0, 91.9] |  | 292 | 90.1 [86.4, 92.9] |  | 301 | 91.2 [87.7, 93.8] |
| Sensoric |  | 67 | 20.9 [16.8, 25.6] |  | 58 | 17.9 [14.1, 22.4] |  | 70 | 21.5 [17.4, 26.3] |  | 74 | 22.8 [18.6, 27.7] |  | 90 | 27.3 [22.7, 32.3] |
| Renal |  | 14 | 4.4 [2.6, 7.2] |  | 12 | 3.7 [2.1, 6.4] |  | 13 | 4.0 [2.4, 6.7] |  | 13 | 4.0 [2.4, 6.7] |  | 12 | 3.6 [2.1, 6.2] |
| Respiratory |  | 52 | 16.2 [12.6, 20.6] |  | 81 | 25 [20.6, 30.0] |  | 80 | 24.6 [20.2, 29.6] |  | 97 | 29.9 [25.2, 35.1] |  | 119 | 36.1 [31.1, 41.4] |
| Dysphagic |  | 32 | 10.0 [7.2, 13.7] |  | 33 | 10.2 [7.3, 14.0] |  | 40 | 12.3 [9.2, 16.3] |  | 46 | 14.2 [10.8, 18.4] |  | 48 | 14.5 [11.1, 18.8] |
| Muscular |  | 182 | 56.7 [51.2, 62.0] |  | 155 | 47.8 [42.5, 53.3] |  | 162 | 49.8 [44.4, 55.3] |  | 149 | 46.0 [40.6, 51.4] |  | 193 | 58.5 [53.1, 63.7] |
| Walk |  | 109 | 34.0 [29.0, 39.3] |  | 95 | 29.3 [24.6, 34.5] |  | 108 | 33.2 [28.3, 38.5] |  | 119 | 36.7 [31.7, 42.1] |  | 153 | 46.4 [41.1, 51.8] |
| Mis- or malsensation |  | 94 | 29.3 [24.6, 34.5] |  | 104 | 32.1 [27.3, 37.4] |  | 131 | 40.3 [35.1, 45.7] |  | 133 | 41.0 [35.8, 46.5] |  | 146 | 44.2 [39.0, 49.6] |
| Weight |  | 55 | 17.1 [13.4, 21.6] |  | 39 | 12.0 [8.9, 16.0] |  | 23 | 7.1 [4.8, 10.4] |  | 10 | 3.1 [1.7, 5.6] |  | 10 | 3.0 [1.7, 5.5] |
| Incontinence |  | 34 | 10.6 [7.7, 14.4] |  | 43 | 13.3 [10.0, 17.4] |  | 41 | 12.6 [9.4, 16.7] |  | 46 | 14.2 [10.8, 18.4] |  | 56 | 17.0 [13.3, 21.4] |
| Sexual |  | 27 | 8.4 [5.8, 12.0] |  | 41 | 12.7 [9.5, 16.7] |  | 57 | 17.5 [13.8, 22] |  | 74 | 22.8 [18.6, 27.7] |  | 83 | 25.2 [20.8, 30.1] |
| Pain |  | 70 | 21.8 [17.6, 26.6] |  | 87 | 26.9 [22.3, 31.9] |  | 80 | 24.6 [20.2, 29.6] |  | 97 | 29.9 [25.2, 35.1] |  | 118 | 35.8 [30.8, 41.1] |
| Other |  | 114 | 35.5 [30.5, 40.9] |  | 127 | 39.2 [34.0, 44.6] |  | 101 | 44.9 [39.6, 50.4] |  | 152 | 46.9 [41.5, 52.4] |  | 161 | 48.8 [43.4, 54.2] |
| Fatigue |  | 65 | 20.2 [16.2, 25.0] |  | 88 | 27.2 [22.6, 32.3] |  | 83 | 31.1 [26.3, 36.3] |  | 107 | 33.0 [28.1, 38.3] |  | 122 | 37.0 [31.9, 42.3] |
| Sleep |  | 68 | 21.2 [17.1, 26.0] |  | 67 | 20.7 [16.6, 25.4] |  | 146 | 25.5 [21.1, 30.5] |  | 83 | 25.6 [21.2, 30.6] |  | 85 | 25.8 [21.3, 30.7] |

N = number of patients included in the assessment; n = number of patients reporting the respective impairment; % = proportion of patients reporting the respective impairment; CI = confidence interval.

## Table S8: Predictors of activities of daily living (ADL) score at one- and three-year post-sepsis assessed in one- and three-year sepsis survivors from the simple linear regression analysis

| **Predictors** [Reference category] | **1 year post-sepsis** (n = 459) | | |  | **3 years post-sepsis** (n=330) | | |
| --- | --- | --- | --- | --- | --- | --- | --- |
|  | **β** | **95% CI for β** | **p-value** |  | **β** | **95% CI for β** | **p-value** |
| Age | -0.163 | [-0.252, -0.073] | <0·001 |  | -0.235 | [-0.396, -0.075] | 0.004 |
| Sex [male] | -0.102 | [-0.193, -0.011] | 0.027 |  | -0.354 | [-0.814, 0.107] | 0.132 |
| Maximum SOFA score | -0.071 | [-0.164, 0.022] | 0.133 |  | -0.133 | [-0.521, 0.255] | 0.501 |
| Renal failure [absence] | -0.113 | [-0.204, -0.022] | 0.015 |  | -0.325 | [-1.178, 0.527] | 0.455 |
| Chronic cardiovascular disease [absence] | -0.049 | [-0.141, 0.042] | 0.291 |  | -0.213 | [-0.645, 0.220] | 0.335 |
| Cancer [absence] | 0.070 | [-0.022, 0.161] | 0.137 |  | -0.051 | [-0.541, 0.440] | 0.839 |
| Chronic cerebrovascular disease [absence] | -0.022 | [-0.114, 0.070] | 0.641 |  | 0.536 | [-0.510, 1.582] | 0.315 |
| Liver disease [absence] | 0.101 | [0.010, 0.192] | 0.030 |  | 0.199 | [-0.554, 0.953] | 0.604 |
| Chronic respiratory disease [absence] | -0.033 | [-0.126, 0.059] | 0.479 |  | 0.156 | [-0.505, 0.817] | 0.643 |
| Dementia [absence] | -0.159 | [-0.249, -0.07] | 0.001 |  |  |  |  |
| Pre-sepsis ADL score | 0.455 | [0.380, 0.530] | <0·001 |  | 0.712 | [0.551, 0.872] | <0·001 |
| Number of organ replacement therapies | 0.055 | [-0.037, 0.147] | 0.238 |  | -0.047 | [-0.348, 0.254] | 0.759 |
| Length of ICU stay | -0.144 | [-0.234, -0.054] | 0.002 |  | -0.023 | [-0.111, 0.065] | 0.603 |
| Discharge to rehab [absence] | -0.084 | [-0.175, 0.007] | 0.072 |  | -0.157 | [-0.659, 0.344] | 0.539 |

Predictors were assessed at the index ICU stay except for discharge disposition (assessed at hospital discharge) and pre-sepsis ADL score (assessed at three-months follow-up). We treated ADL score (range 0 - 100), age and maximum SOFA score (range 0 - 24), number of organ replacement therapies, length of ICU stay as metric and the remaining variables as binary. β = standardized regression coefficients, CI = confidence interval. n = number of analyzed survivors. For three-year post-sepsis, dementia was a constant because none of the included sepsis survivors suffered from dementia. Reading example: The standardized regression coefficient β for the predictor “pre-sepsis ADL score” corresponds to the change in the ADL score, when the pre-sepsis ADL score changes by one standard deviation

**References**

1. Mahoney FI, Barthel DW. FUNCTIONAL EVALUATION: THE BARTHEL INDEX. *Md State Med J* 1965; **14**: 61-5.

2. Deppermann K-M, Friedrich C, Herth F, Huber RM. Geriatrische Assessments und Diagnostik beim älteren Patienten. *Onkologie* 2008; **31**: 9.

3. Katz MJ, Wang C, Nester CO, et al. T-MoCA: A valid phone screen for cognitive impairment in diverse community samples. *Alzheimers Dement (Amst)* 2021; **13**(1): e12144.

4. Ehrensperger MM, Berres M, Taylor KI, Monsch AU. Screening properties of the German IQCODE with a two-year time frame in MCI and early Alzheimer's disease. *Int Psychogeriatr* 2010; **22**(1): 91-100.

5. Siegrist P, Maercker A. Deutsche Fassung der Short Screening Scale for DSM-IV Posttraumatic Stress Disorder. Aktueller Stand der Validierung. 2010; 2010.

6. Breslau N, Peterson EL, Kessler RC, Schultz LR. Short screening scale for DSM-IV posttraumatic stress disorder. *Am J Psychiatry* 1999; **156**(6): 908-11.

7. Derogatis L. BSI-18. Brief Symptom Inventory-18. Administration, Scoring, and Procedures Manual. . Minneapolis: MN: NCS Pearson, INC; 2000.

8. Jackson C. The Chalder Fatigue Scale (CFQ 11). *Occup Med (Lond)* 2015; **65**(1): 86.

9. Rabin R, de Charro F. EQ-5D: a measure of health status from the EuroQol Group. *Ann Med* 2001; **33**(5): 337-43.

10. Jackson CH. Multi-State Models for Panel Data: The msm Package for R. *J Stat Softw* 2011; **38**(8): 1-28.

11. Haines KJ, Hibbert E, McPeake J, et al. Prediction Models for Physical, Cognitive, and Mental Health Impairments After Critical Illness: A Systematic Review and Critical Appraisal. *Crit Care Med* 2020; **48**(12): 1871-80.

12. Rosseel Y. lavaan: An R Package for Structural Equation Modeling. *J Stat Softw* 2012; **48**(2): 1 - 36.
